# Honeydew-associated microbes elicit defense responses against brown planthopper in rice

**DOI:** 10.1093/jxb/erz041

**Published:** 2019-03-02

**Authors:** David Wari, Md Alamgir Kabir, Kadis Mujiono, Yuko Hojo, Tomonori Shinya, Akio Tani, Hiroko Nakatani, Ivan Galis

**Affiliations:** 1 Institute of Plant Science and Resources, Okayama University, Kurashiki, Japan; 2 Faculty of Agriculture, Mulawarman University, Samarinda, Indonesia

**Keywords:** Honeydew-associated microorganisms, phytoalexins, plant defense, rice (*Oryza sativa*), rice brown planthopper (*Nilaparvata lugens*), sucking insect

## Abstract

Feeding of sucking insects, such as the rice brown planthopper (*Nilaparvata lugens*; BPH), causes only limited mechanical damage on plants that is otherwise essential for injury-triggered defense responses against herbivores. In pursuit of complementary BPH elicitors perceived by plants, we examined the potential effects of BPH honeydew secretions on the BPH monocot host, rice (*Oryza sativa*). We found that BPH honeydew strongly elicits direct and putative indirect defenses in rice, namely accumulation of phytoalexins in the leaves, and release of volatile organic compounds from the leaves that serve to attract natural enemies of herbivores, respectively. We then examined the elicitor active components in the honeydew and found that bacteria in the secretions are responsible for the activation of plant defense. Corroborating the importance of honeydew-associated microbiota for induced plant resistance, BPHs partially devoid of their microbiota via prolonged antibiotics ingestion induced significantly less defense in rice relative to antibiotic-free insects applied to similar groups of plants. Our data suggest that rice plants may additionally perceive herbivores via their honeydew-associated microbes, allowing them to discriminate between incompatible herbivores—that do not produce honeydew—and those that are compatible and therefore dangerous.

## Teaser

The digestive waste product of sucking herbivores, known as honeydew, betrays the presence of brown planthopper to rice plants, due to the bacteria contained in the honeydew secretions.

## Introduction

The rice brown planthopper (BPH), *Nilaparvata lugens* (Stål) (Hemiptera: Delphacidae), is one of the most serious pests of rice in Asia. It is alarming that a sharp increase in the frequency and extent of hopper outbreaks has been noticed since ~2002, resulting in gross losses of rice crop, particularly in China, Thailand, and Vietnam (Fujita *et al.*, 2013). Rice damage (often called ‘hopper burn’ as opposed to viral symptoms) results from extensive feeding of hoppers, and extraction of phloem sap by means of a stylet protected by a salivary sheath (Wang *et al.*, 2008). Saliva is secreted into phloem which facilitates hopper feeding and interferes with the plant’s immune system (Petrova and Smith, 2015; Ji *et al.*, 2017; Ye *et al.*, 2017). Due to ingestion of assimilate-rich sap, large amounts of sticky feeding residue, known as honeydew (Auclair, 1963), are deposited on plants through feeding of compatible insects. Honeydew then supports growth of various microbes on the plant surface, leading to a sooty appearance of infested plants (Fujita *et al.*, 2013; Supplementary Fig. S1A at *JXB* online).

The broadly accepted concept of plant–herbivore co-evolution assumes that plants attacked by a diverse community of insects and pathogens evolve an equally diverse set of immune responses (Spoel and Dong, 2008; Fürstenberg-Haag *et al*., 2013). This implies that plants should also evolve diverse recognition mechanisms to be able to utilize these different responses adequately. Indeed, similar to plant–pathogen interactions, where plant immunity is triggered by pathogen- or microbe-associated molecular patterns, hereafter referred as PAMPs or MAMPs, respectively (Jones and Dangl, 2006; Zipfel, 2014), several herbivore-associated molecular patterns (HAMPs) have also been found (Felton and Tumlison, 2008; Mithöfer and Boland, 2008; Acevedo *et al.*, 2015). However, in contrast to PAMPs, no receptors for HAMPs have been reported so far. In general, HAMPs occur in oral secretions, saliva, oviposition fluids, and feces of insect herbivores (Rayapuram and Baldwin, 2007). In sucking herbivores, honeydew represents another potential source of HAMPs, but it has not been investigated in detail. However, as honeydew can only be produced by actively feeding insects, this information could be important for plants in order to distinguish compatible and incompatible sucking insect attacks.

Sucking herbivores, including cicadas, aphids, whiteflies, planthoppers, and leafhoppers, transmit viruses, mycoplasma-like organisms, and pathogenic bacteria to plants (Sugio *et al.*, 2011; Deng *et al.*, 2013; Whitfield *et al.*, 2015). In addition to pathogens, they also harbor obligate and/or facultative microbial symbionts inside their body that can be externalized via insect secretions, namely saliva and honeydew (Douglas, 2015; Skidmore and Hansen, 2017). For example, a set of 11 putative proteins from *Buchnera aphidicola* was found in the saliva secreted by potato aphids (*Macrosiphum euphorbiae*) (Chaudhary *et al.*, 2014). Pea aphid (*Acyrthosiphon pisum*) honeydew also contained proteins from the endosymbiotic bacteria and gut flora (Sabri *et al.*, 2013). In addition, sucking herbivores may ingest, together with phloem sap, various microbes from plants (Bove and Garnier, 2002), and secrete them afterwards as part of the honeydew. Finally, insects carry specific and/or non-specific microbes on their surface (Toledo *et al.*, 2011), which may contaminate and further amplify in the nutrient-rich honeydew secretions on plants (Fujita *et al.*, 2013).

Widespread microbial presence in plant–herbivore interactions indicates an important role for microbes in plant defense against herbivores (Kaloshian and Walling, 2016; Schausberger, 2018). Chaperonin GroEL from *Buchnera* species, obligate mutualists and primary endosymbionts of aphids (Wilson *et al.*, 2010), is secreted in saliva and activates plant immune responses (Chaudhary *et al.*, 2014). In another example, the facultative symbiont *Hamiltonella defensa* of whiteflies (*Bemisia tabaci*), secreted through saliva, exploits the antagonistic relationship between salicylic acid (SA) and jasmonic acid (JA) signaling (Glazebrook, 2005) to suppress plant defenses, and thereby benefit the insect host (Su *et al.*, 2015). Similar to sucking herbivores, chewing insect Colorado potato beetle (*Leptinotarsa decemlineata*) larvae utilize bacteria in their oral secretions to manipulate tomato (*Solanum lycopersicum*) defense (Chung *et al.*, 2013*a*, *b*). Bacteria from the oral secretions of the fall armyworm (*Spodoptera frugiperda)* showed various effects on defense in tomato and maize (Acevedo *et al.*, 2017), and bacteria orally secreted by the false potato beetle (*Leptinotarsa juncta)* triggered distinct defense responses in preferred and non-preferred host plants (Wang *et al.*, 2016).

In our research, a strong elicitor activity associated with the honeydew of BPHs, and particularly its macromolecular fraction, was observed. We hypothesized that some of the honeydew-associated microbes could be perceived by rice that triggers stronger defense against BPHs (Alamgir *et al.*, 2016). Reports that planthopper-elicited defenses in rice partially resemble those induced by pathogens further supported this idea (Zhou *et al.*, 2009; Duan *et al.*, 2014; Li *et al.*, 2017). We then isolated seven strains of culturable microbes from BPH honeydew, and investigated their role in rice defense against BPHs. Simultaneously, we prepared microbe-suppressed BPHs to show that honeydew microbes, indeed, betray BPHs to rice, which then mounts stronger immune responses, namely accumulation of more phytoalexins, and potentially recruits more natural enemies of BPHs through the modulation of their volatile emissions.

## Materials and methods

### Plant cultivation and insect rearing

Rice plants (*Oryza sativa* L. var. Nipponbare) were used for insect rearing and collection of honeydew. Seeds were germinated in a nutrient-rich soil Kumiai Ube Baido No.2 (MC Ferticom, http://www.mcferticom.jp/index.html). After 10 d, seedlings were transferred to larger pots with paddy field soil mixed in a 5:1 (v/v) ratio with nutrient-rich substrate as specified above. The plants were kept at 24–26 °C day/20–22 °C night temperatures and ambient humidity at a 14–16 h photoperiod in the growth room supplemented with both natural and fluorescent lights. A colony of Koshi (Kumamoto Prefecture, Japan) field-collected BPHs (*N. lugens*) has been maintained in the laboratory since 2014 on a constant supply of young rice seedlings produced from non-sterile rice seeds, collected annually in the field. Seedlings were germinated and grown in the growth room using commercial soil substrate for rice (OK Soil; ISEKI & CO., Ltd, http://www.iseki.co.jp/english/).

### Honeydew collection

**Fig. 1.**
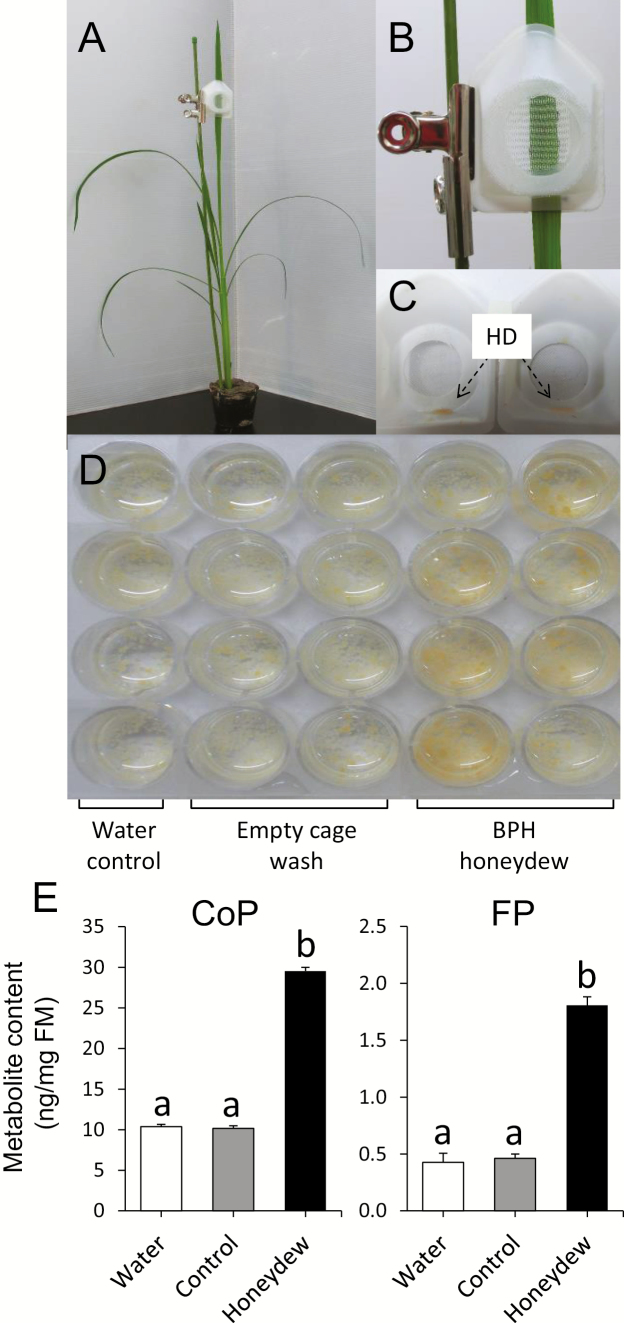
Induction of phytoalexins with honeydew collected from brown planthoppers (BPHs) in cultured rice cells. (A) Honeydew samples were collected from Nipponbare plants infested with 10 BPHs enclosed in a small clip cage (B, C). (D) Examples of change in rice cells and medium induced by honeydew during a 24 h cultivation period. (E) Phenolamide contents in honeydew-treated and control rice cells from individual honeydew collections determined by LC-MS/MS after 24 h treatment. Different letters show statistically significant differences between treatments by ANOVA (*P*<0.05, Tukey HSD test). *n*=4–8; error bars=SE; FM, fresh mass; CoP, *p-*coumaroylputrescine; FP, feruloylputrescine; HD, honeydew.

Ten adult BPH insects were enclosed in a clean 4×6 cm clip cage, which was then attached to a single young leaf of the 6- to 8-week-old rice plant (Fig. 1A, B) kept in the growth room under controlled temperature conditions. Typically, 96 h later, honeydew deposits on the clip cages as shown in Fig. 1C were carefully collected using a 20 μl pipette tip, with a small amount of sterile water as diluent, and transferred into sterile microcentrifuge tubes. An identical procedure was used to wash the surface of clip cages attached to plants without BPHs, which was then used for control treatments. All collected fractions were stored at –80 °C until used in experiments (cell assays, intact plant treatments, microbe isolations, and identifications).

### Isolation and cultivation of BPH honeydew-associated microbes

Honeydew from BPHs was plated in dilution series on agar plates with culture medium, LB Broth, Lennox (Nacalai Tesque, Kyoto, Japan) or Gifu Anaerobic Medium (GAM; Nissui Pharmaceutical Co., Ltd, Tokyo, Japan), and incubated for 2 d at 28 °C. Single colonies from plates with distinct morphologies were purified through several passages, and isolated microbial strains were streaked onto a master plate for maintenance, identification, and characterization.

### Elicitation of rice cells

Cultured cells were used to examine the effect of honeydew extracts and microbes on induced defense responses of rice. Cell suspensions were derived from mature rice seed embryos placed on a modified N6 callus-forming agar medium supplemented with 1 mg l^–1^ synthetic auxin (2,4-D). Cells were transferred and propagated in 300 ml conical flasks containing liquid N6 culture medium under constant shaking (120 rpm) at 25 °C, and passage-propagated weekly as described previously in Shinya *et al.* (2016). Before each experiment, 40 mg aliquots of freshly transferred cells were placed in a 24-well microtiter plate (Techno Plastic Products AG, Switzerland) and pre-incubated in 1 ml of new culture medium for 30 min to subdue initial stress responses. Equivalent amounts of honeydew or water from empty clip cage washes were directly applied to treatment and mock control groups, respectively. Bacterial isolates from honeydew, grown on LB plate for 2 d at 28 °C, were suspended in sterile water, adjusted to OD_600_=0.2, and then 2 μl aliquots were added to pre-incubated cells. Pure water and 10 nM chitin oligomer (GlcNAc)_8_ were used as negative and positive control treatments, respectively (Shinya *et al.*, 2016). Treated cells were incubated at slow speed on a Taitec MicroMixer E-36 (Taitec Corporation, Saitama, Japan) for 24 h under growth conditions described for plants. Harvested cells, after removal of liquids by micropipette, were frozen in liquid nitrogen and stored at –80 °C until analysis.

### Treatment of intact rice plants

Rice seeds (*O. sativa* var. Nipponbare) were germinated as described above, and cultivated for ~6 weeks, after which the last fully developed leaf (~20×1 cm) was used for treatments. Typically, 2 μl of concentrated honeydew collection (or the respective control solution) were applied on the leaf, and gently rubbed on the surface with fingers covered by a clean rubber glove. To mimic BPH herbivory that includes small piercing wounds, the last fully developed leaf was wounded with a fabric pattern wheel along the midvein, and wounds were immediately treated with 2 μl of concentrated honeydew, or the respective control solution. Representative microbial isolates were suspended as described for cell treatments but using 15% (w/v) sucrose in sterile water, and 2–5 μl aliquots were rubbed on intact or wounded leaves (sucrose was used as control). For real herbivory, 10 BPH adults were applied to the last fully developed leaf enclosed in 4×6 cm clip cages. Treated parts of the leaves were sampled at 24, 48, and 72 h time points after treatment, immediately frozen in liquid nitrogen, and kept at –80 °C until analysis.

### Classification of BPH-associated microbes by MALDI-TOF/MS

Microbes isolated from honeydew were subjected to matrix-assisted laser desorption/ionization-time of flight/MS (MALDI-TOF/MS) analysis as described in Tani *et al.* (2012), with some modifications. Using a toothpick, bacterial cells were lifted from the master plates and spotted onto the MALDI steel target plate, and dried in air. Then, 2 µl of matrix solution (saturated solution of sinapinic acid in 50% acetonitrile and 2.5% trifluoroacetic acid) was overlaid onto each sample, and samples were allowed to dry in air. The samples were analyzed with MALDI-TOF/MS equipped with a 50 Hz nitrogen laser (Ultraflex, Bruker Daltonics Inc., Billerica, MA, USA). Mass spectra were recorded using a positive linear mode in a range of *m/z* 2000–20 000 with suppression 800 Da (parameter settings: ion source 1, 25 kV; ion source 2, 23.35 kV; lens, 6.35 kV; detector gain, 8.4×). Protein standard was composed of insulin ([M+H]^+^=5734.56), ubiquitin-I ([M+H]^+^=8565.89), cytochrome *c* ([M+H]^+^=12361.09 and [M+2H]^2+^=6181.05), and myoglobin ([M+H]^+^=16952.55 and [M+2H]^2+^=8476.77) (Bruker Daltonics Inc.). The laser shots were applied until the intensity (arbitrary unit) of the highest peak reached between 6000 and 10 000 (usually 300–1000 shots). *Escherichia coli* DH5α (a derivative of *E. coli* K12) was used as a standard to validate the method. The data were analyzed with MALDI BioTyper 3.0 software (Bruker Daltonics Inc.) to construct a main spectra projection (MSP) dendrogram based on spectra similarity using default program settings as described in Tani *et al.* (2015).

### Identification of honeydew microbes by DNA sequencing

Representative isolates in the MSP dendrogram were subjected to 16S rRNA gene sequencing. Genomic DNA was extracted from isolated colonies of representative strains and direct PCR was used to amplify ~1.5–kb 16S rRNA gene fragments from the genomic DNA using the fD1 and rD1 primer set as reported by Weisburg *et al.* (1991). The PCR conditions were 30 cycles of 30 s at 94 °C, 30 s at 52 °C, and 2 min at 72 °C, with subsequent final extension at 72 °C for 7 min. An exception applied to isolate 4-24, which only amplified at the annealing temperature of 54 °C. Direct sequencing of amplified PCR products with the original fD1 and rD1 primers was conducted using an ABI Prism BigDye Terminator v3.1 Cycle Sequencing Kit (Applied Biosystems, Carlsbad, CA, USA) on a 3130/3130*xl* Genetic Analyzer (Applied Biosystems, Foster City, CA, USA). Furthermore, selected PCR products were re-amplified, size-fractionated on an agarose gel, excised, purified, and cloned into the pGEM-T Easy vector system (Promega Corp., Madison, WI, USA) for high quality sequencing with vector-specific primers. Nucleotide sequences were analyzed using Genetyx ver. 11 (Genetyx Corp., Tokyo, Japan). Nucleotide BLAST and homology search against registered 16S rRNA gene nucleotide sequences in the EzBioCloud database (Yoon *et al.*, 2017) was conducted to identify microbes. The EzBioCloud results were cross-referenced with the user-deposited nucleotide sequences of 16S rRNA genes in the DDBJ (www.ddbj.nig.ac.jp) and EMBL (www.ebi.ac.uk) databanks.

### Antibiotic resistance of microbes and development of microbe-free BPHs

Antibiotic resistance for each microbial isolate was tested using 11 different commercially supplied antibiotics: ampicillin (50 µg ml^–1^), carbenicillin (50 µg ml^–1^), kanamycin (50 µg ml^–1^), chloramphenicol (20 µg ml^–1^), rifampicin (50 µg ml^–1^), tetracycline (15 µg ml^–1^), gentamicin (50 µg ml^–1^), nalidixic acid (30 µg ml^–1^), neomycin (10 µg ml^–1^), spectinomycin (50 µg ml^–1^), and streptomycin (50 µg ml^–1^). Microbial isolates were applied on LB plates with antibiotics using a sterile toothpick and examined after growing for 2 d at 28 °C. Antibiotics effective against microbes were combined and used for treatment of BPHs as shown in Supplementary Fig. S10. A clean 50 ml plastic tube containing 20 ml of sterile water (control treatment) or antibiotic cocktail (test treatment) was used with a bunch of 10–15 cm long stem cuttings freshly removed from the 2- to 3 week-old rice seedlings. Stems were inserted via a 5 mm hole punctured in the lid, and cut surfaces were immediately submerged in sterile water or antibiotic cocktail. To allow collection of clean honeydew from water- and antibiotic-treated BPHs, a Parafilm (BEMIS, Neenah, WI, USA) layer was placed on top of each lid, closely surrounding the rice stems (Supplementary Fig. S10). Honeydew released from BPH by gravity was collected from the Parafilm, and used to determine microbial presence on LB plates using serial dilutions. Seedlings and antibiotics were replaced daily for 4 d, after which the antibiotic concentration was decreased to one-fifth, and BPHs were allowed to partially recover for another 2 d on the plants. BPHs were then captured, after being briefly anesthetized with carbon dioxide, and placed in larger 9×9 cm clip cages for plant treatments with 4–9 biological replicates, using set-up and time periods as described in each experiment.

### BPH performance tests

In experiments shown in Supplementary Fig. S11A, the youngest developed leaf on 6-week-old rice plants was wounded and 5 µl of crude or filtered (0.22 µm pore) honeydew was immediately applied by rubbing on the surface. BPHs (10 adults or 15 nymphs at 3–4 instars) were applied 24 h later to the upper part of the leaf inserted through a slit in a plastic cylinder (8 cm diameter, 25 cm length; Supplementary Fig. S11D). Dead BPHs were counted after 7 d. In experiments shown in Supplementary Fig. S11B and C, 10 µl of crude or filtered honeydew was directly applied by rubbing on intact leaves. BPHs (15 nymphs at 3–4 instars) were applied to the plants after 3 d, as described above. Dead BPHs were counted at 2, 4, and 6 d after feeding exposure.

### Quantification of secondary metabolites

The protocol described in Tanabe *et al.* (2016) was followed. Cells or liquid nitrogen-pulverized leaves were suspended in Extraction Buffer 1 [40% (v/v) methanol in 84 mM ammonium acetate buffer, pH 4.8]. After addition of ceramic beads (BMS, Tokyo, Japan), samples were homogenized for 45 s in a ball mill FastPrep 24 (MP Biomedicals, Santa Ana, CA, USA) adjusted to a pre-set grinding level of 5.0. Suspensions were centrifuged at 16 000 *g*, 4 °C for 15 min, and cleared supernatants were transferred into 2 ml microcentrifuge tubes. Pellets were re-extracted with Extraction Buffer 2 [80% (v/v) methanol in 84 mM ammonium acetate buffer, pH 4.8], vigorously mixed at room temperature in a shaker for 10 min, and centrifuged as before. Supernatants from both extractions were combined, diluted with 84 mM ammonium acetate buffer, pH 4.8 to a final 20% (v/v) methanol concentration, and loaded on pre-conditioned solid phase extraction columns (3 ml size, Bond Elut-C18, 200 mg, Agilent Technologies, Santa Clara, CA, USA). After brief drying with an air purge from the attached syringe, samples were slowly eluted with 1.5 ml of 100% methanol (Wako Pure Chemical Industries, Ltd, Osaka, Japan). After brief clearing of eluates in a microcentrifuge at maximum speed, 10 μl aliquots were measured on a triple quadrupole LC-MS/MS 6410 system (Agilent Technologies) equipped with a Zorbax SB-C18 column (50×2.1 mm ID, 1.8 μm, Agilent Technologies), essentially as described in Alamgir *et al.* (2016).

### Collection of headspace volatile organic compounds (VOCs)

A headspace collection system was used as described previously in Sobhy *et al.* (2017). Independent rice plants in pots (4–6 weeks old) were treated on the last fully developed leaf with 10 BPHs, 2 μl or 5 μl of concentrated honeydew, or wounding combined with honeydew application. Control leaves were used without treatments but still covered with empty clip cages where appropriate. For each collection, plants grown in a pot covered in a plastic Ziploc bag to limit soil volatiles were inserted in an acrylic cylinder (67 cm high×10 cm ID), and each cylinder with inlet and outlet ports was flushed with air at ~0.75 l min^−1^, passing through 10 cm glass traps (5 mm ID) containing Porapak Q sorbent (200 mg, Supelco Analytical, Bellefonte, PA, USA). In every experiment, a set of 12 cylinders was connected to a single vacuum pump ULVAC DAP-12S (ULVAC KIKO Inc., Japan), while an additional four traps were used for collection and detection of background air volatiles. The bottom part of each cylinder was placed in 5 cm of water for complete closure of the system. VOCs were collected in 24 h periods for up to 2–3 d, depending on the experiment. Samples were eluted from traps with 1 ml of dichloromethane, spiked with 400 ng of tetralin (1,2,3,4-tetrahydronaphthalene; Nacalai Tesque, Kyoto, Japan) as an internal standard, and analyzed on a 240 Ion Trap Mass Spectrometer coupled to an Agilent 7891A GC using settings and conditions described in Sobhy *et al.* (2017). MS data collected in the mass range *m/z* 40–300 were analyzed by Agilent Workstation 7.02. Tentative identifications were made by comparison of spectra with the NIST 2011 Mass Spectral Library and Software (US National Institute of Standards and Technology, USA), and confirmed by co-elution with authentic standards (linalool, limonene, and caryophyllene; Wako Pure Chemical Industries, Ltd). Linalool, limonene, and caryophyllene contents were quantified using external standard calibration curves.

### Phytohormone quantifications

Rice leaves (30–100 mg fresh mass) treated with honeydew or isolated microbes were harvested and flash-frozen in liquid nitrogen. Phytohormone extraction and analysis was performed essentially as described by Fukumoto *et al.* (2013), using deuterium-labeled internal standards available for JA, jasmonoyl-l-isoleucine (JA-Ile), abscisic acid (ABA), and SA. OPDA (12-oxo-phytodienoic acid), for which the authentic deuterated standard was not available, was quantified using the structurally related internal standard d3-JA, and expressed as equivalent of the respective compound.

### Statistical analyses

Statistical analyses (one way ANOVA) were carried out with the open source software OpenStat (http://openstat.info/OpenStatMain.htm) or a commercial version of Microsoft Excel (Microsoft Corporation, Redmond, WA, USA; Student’s *t*-test).

## Results

### Elicitation of rice secondary metabolism with honeydew

**Fig. 2.**
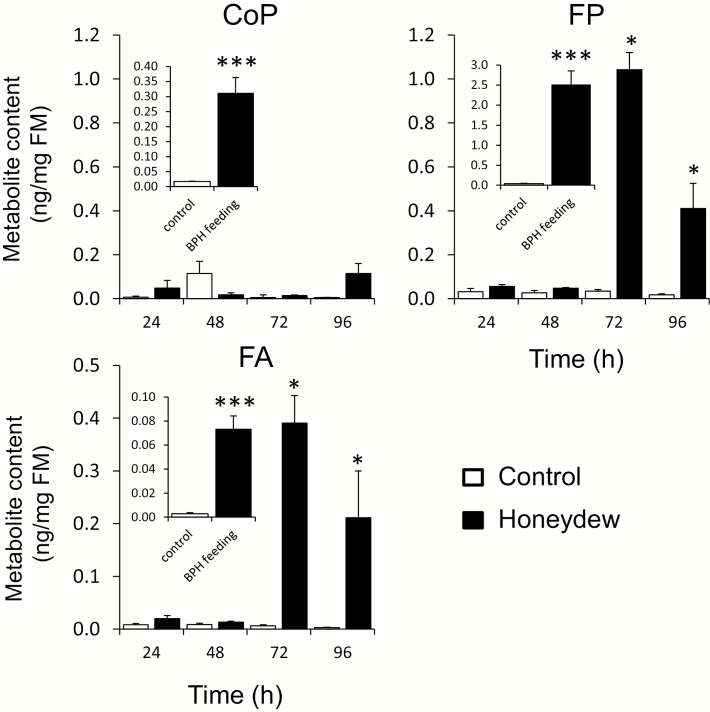
Accumulation of phytoalexins in rice leaves treated with honeydew. Metabolite content was measured in the leaves every 24 h by LC-MS/MS up to 96 h. Insets in each graph show induced metabolite levels in rice plants subjected to BPH feeding for 72 h. Asterisks indicate statistically significant differences between treatment and control of the respective time point, determined by Student’s *t*-test (*P<0.05; ***P<0.001). *n*=3; error bars=SE; FM, fresh mass.

To determine the effects of the sucking pest’s honeydew on rice defense, honeydew was collected from the BPHs kept on *O. sativa* L. var. Nipponbare plants, using a clip cage with 10 adult insects feeding on a leaf (Fig. 1A–C). In *in vivo* bioassays, rice cultured cells treated with honeydew isolates showed a strong variation in cell and media color between control (empty-cage-wash) and individual honeydew isolates (Fig. 1D). We assumed that BPH secretions have triggered specific changes in rice secondary metabolism, and therefore harvested the cells and examined their secondary metabolite levels. Two rice phenolamides, *p*-coumaroylputrescine (CoP) and feruloylputrescine (FP), were strongly induced at 24 h after addition of 2 μl of crude BPH honeydew to 1 ml of cultivation medium with cells (Fig. 1E; Supplementary Fig. S1). To confirm that these phytoalexins are also induced in intact plants, the last fully developed leaves of the 6-week-old rice seedlings were treated with 2 μl of crude honeydew, which was gently rubbed on the leaf surface. While metabolite induction was not observed at 24 h and 48 h post-treatment, FP and another herbivory-induced leaf phytoalexin, feruloylagmatine (FA), accumulated more at 72 h post-treatment compared to control leaves (Fig 2; Supplementary Fig. S2). Similar to honeydew treatments, the levels of CoP, FP, and FA were induced by BPH feeding after 72 h (insets in Fig. 2).

### BPH honeydew induces rice VOCs

**Fig. 3.**
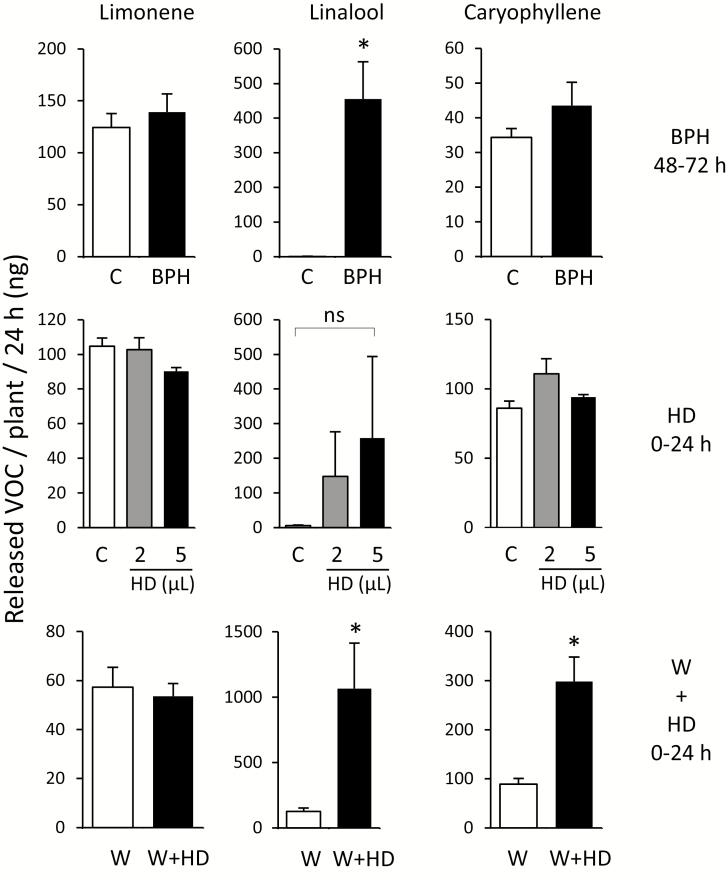
Volatile organic compounds released from honeydew-treated leaves. Monoterpenes (limonene and linalool) and sesquiterpene (caryophyllene) were determined by GC-MS in the headspace of rice plants treated with BPH infestation, plants treated on the last fully developed leaf with 2 µl or 5 µl of concentrated honeydew, and plants treated on the last fully developed leaf with wounding using a serrated fabric pattern wheel, followed by application of 2 µl of honeydew. Asterisks indicate statistically significant differences between treatments determined by ANOVA (honeydew treatment, no significant changes found) or Student’s *t*-test (*P<0.05). C, control untreated plants; W, wounding; HD, honeydew; *n*=4–6; error bars=SE.

Limonene, linalool, and caryophyllene are typical volatiles released from rice plants, constitutively (limonene and caryophyllene) and/or after herbivory (linalool and caryophyllene) (Gomez *et al.*, 2005; Mithöfer and Boland, 2012; Sobhy *et al.*, 2017). When 6-week-old rice seedlings were exposed to feeding of BPHs, release of monoterpene linalool to the headspace was promoted, but monoterpene limonene and sesquiterpene caryophyllene levels remained unchanged. While direct rubbing of 2 μl or 5 μl of honeydew on the leaf did not induce significant changes in VOC emissions, linalool levels increased in a dose-dependent manner after honeydew application (Fig. 3). Next, we made a series of small wounds to the leaves, using a serrated fabric pattern wheel, and immediately applied honeydew that resembled honeydew deposition on the BPH-pierced leaf surface that, under natural conditions, also includes mechanical abrasions, chewing damage, and necrotic lesions (Supplementary Fig. S1A). This time, significantly higher emissions of linalool and caryophyllene were observed (Fig. 3), suggesting that honeydew indeed amplifies rice indirect defense responses in wounded leaves, which may also apply to field-grown rice plants.

### Filtered honeydew shows reduced activity in rice cells

**Fig. 4.**
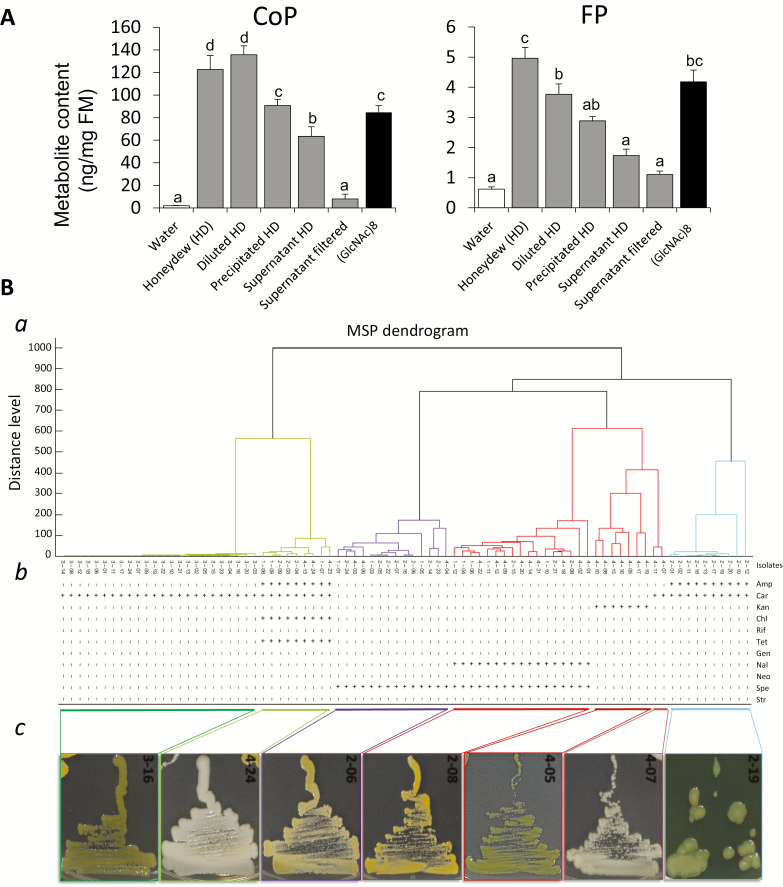
Induction of phytoalexins in rice cells treated with raw and filtered BPH honeydew and bacterial isolates from honeydew. (A) Phenolamide contents in cells treated with raw or processed honeydew determined by LC-MS/MS after 24 h treatment. Chitin oligomer (GlcNAc)_8_ was used as positive control. Different letters show statistically significant differences between treatments by ANOVA (*P*<0.05; Tukey HSD test). *n*=3; error bars=SE; FM, fresh mass. (B) (a) Biotyper-generated MSP dendrogram based on the protein mass spectra. Different tree colors suggest a species-level identification (isolates under a tree of the same color are likely to belong to the same species). (b) Antibiotic resistance of microbial symbionts isolated from the BPH honeydew. ‘+’ is used for resistant, and ‘–’ for sensitive colonies on LB plates. Antibiotics are Amp, ampicillin (50 µg ml^–1^); Car, carbenicillin (50 µg ml^–1^); Kan, kanamycin (50 µg ml^–1^); Chl, chloramphenical (20 µg ml^–1^); Rif, rifampicin (50 µg ml^–1^); Tet, tetracycline (15 µg ml^–1^); Gen, gentamicin (50 µg ml^–1^); Nal, nalidixic acid (30 µg ml^–1^); Neo, neomycin (10 µg ml^–1^); Spe, spectinomycin (50 µg ml^–1^); and Str, streptomycin (50 µg ml^–1^). (c) Images of the representative microbial isolates from each combined MSP/antibiotic resistance clade.

The strong induction of rice phytoalexins in both rice cells and seedlings, and VOCs in the seedlings suggested that honeydew contains a dominant elicitor of rice defense. In order to size-fractionate honeydew, and separately examine elicitor activity in each fraction, we centrifuged honeydew after dilution in sterile water 1:10 (v/v) at 15 000 *g* for 15 min at 4 °C. When the supernatants were tested for elicitor activity, CoP and FP levels were highly reduced compared with application of crude (2 μl) and diluted (10 μl) honeydew, or 10 μl of re-suspended pellet (in 100 μl of sterile water) to rice cells (Fig. 4A; Supplementary Fig. S3). Next, supernatants were passed through a 0.22 µm filter (Syringe-Filter 0.22 µm, TPP Techno Plastic Products AG, Switzerland). When cells were treated with 10 μl of the filtrate, CoP and FP levels were even more reduced compared with centrifuged honeydew treatments (Fig. 4A). Substantial loss of elicitor activity during centrifugation and/or filtration indicated that honeydew from BPH contains a fairly large molecular size elicitor, such as microbial cells, which strongly alters phytoalexin profiles in rice. Such a putative microbial elicitor was stable through a heat treatment of honeydew at 100 °C for 20 min (Supplementary Fig. S4). Therefore, although we refer to elicitors in honeydew as microbes, the actual elicitors should be heat-stable molecular patterns derived from the bacterial cells, such as hairpin-like proteins, flagellin, or oligosaccharides.

### BPH honeydew contains microbes with diverse antibiotic resistance

The BPH body contains microbial cells, both internally and externally, either of which may eventually appear in honeydew. Although obligate microbes cannot be easily cultured on artificial media, assuming that microbes in honeydew should survive and proliferate on rice leaves, we approached isolation of microbes from BPH honeydew using a simple type of cultivation medium (see the Materials and methods). A total of 84 microbial isolates were obtained from raw BPH honeydew samples collected on four different occasions (designated by prefixes 1–4 in isolate numbers) and maintained on LB medium for further experiments.

At first, whole-cell MALDI-TOF/MS analysis was used to group BPH honeydew-associated microbial isolates into species, based on the similarity of their major protein fingerprints (Fig. 4Ba). In addition, antibiotic resistance was examined with 11 different antibiotics (Fig. 4Bb). Because patterns of antibiotic resistance correlated well with the visual appearance of bacterial colonies (Fig. 4Bc), and the dendrogram, isolates could be divided into seven major clusters, and representative isolates (2-06, 2-08, 2-19, 3-16, 4-05, 4-07, and 4-24) from each cluster were subjected to 16S rRNA gene sequencing. The sequences obtained were compared with EzBioCloud (Table 1), DDBJ, and EMBL databases (Supplementary Table S1). Isolates 2-06, 2-19, and 4-24 showed high sequence similarity (>99%) to those with accession numbers JQ975877, GU124492, and GU124498, respectively, that belong to reported endosymbionts of *N. lugens* (Wang *et al.*, 2015; Supplementary Table S1).

**Table 1.** Characterization of microbes isolated from BPH honeydew

Isolate	Closest match	Similarity (%)	Hit accession	EzBioCloud reference
2-06	*Staphylococcus sciuri*	99.59	AJ421446	Kloos *et al.* (1976)
2-08	*Staphylococcus xylosus*	99.46	D83374	Kloos and Schleifer (1975)
2-19	*Acinetobacter soli*	99.93	APPU01000012	Kim *et al.* (2008)
3-16	*Pantoea dispersa*	99.93	DQ504305	Gavini *et al.* (1989)
4-05	*Microbacterium laevaniformans*	98.64	Y17234	Collins *et al.* (1983)
4-07	*Corynebacterium glycinophilum*	99.24	CP006842	Al-Dilaimi *et al.* (2015)
4-24	*Serratia marcescens* subsp. *marcescens*	99.80	JMPQ01000005	Bizio (1823)

The isolates were identified by search against the EzBioCloud database after amplification of 16S rRNA gene sequences from representative bacterial isolates using the fD1 and rD1 primer combinations as described in the Materials and methods. See Supplementary Table S1 for additional blast hits found in DDBJ/EMBL databanks.

### Some but not all honeydew microbes elicit phenolamide levels in rice

**Fig. 5.**
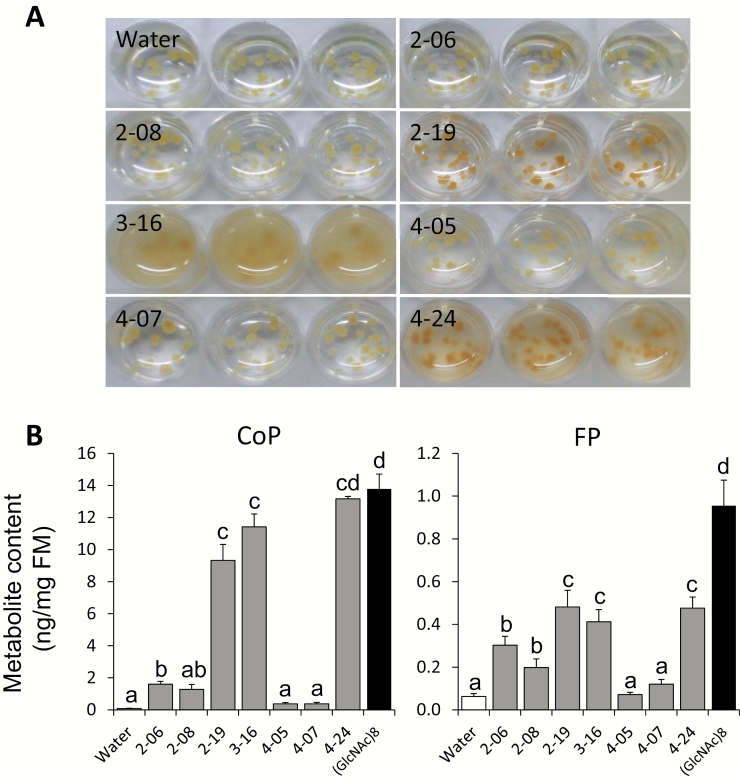
Activity of bacterial isolates in promotion of rice secondary metabolism. (A) Change observed in rice cells inoculated in triplicate with 2 µl of microbial isolates (OD_600_=0.2) from BPH honeydew. (B) Accumulation of phytoalexins in rice cells 24 h after inoculation with 2 µl of microbial isolates (OD_600_=0.2) from BPH honeydew. Chitin oligomer (GlcNAc)_8_ was used as positive control. Different letters show statistically significant differences between treatments by ANOVA (*P*<0.05; Tukey HSD test). *n*=3; error bars=SE; FM, fresh mass.

All seven isolates were examined for their potential to elicit rice defenses. Prominent color changes were observed in rice cells treated with the microbial isolates 2-19, 3-16, and 4-24 (Fig. 5A). After visual assays, metabolites were extracted and phytoalexin levels were determined as before. Cells treated with isolates 2-19, 3-16, and 4-24 showed highly increased levels of CoP and FP relative to water-treated controls (Fig. 5B). Induction levels were comparable with those of chitin oligomer (GlcNAc)_8_, a potent PAMP known to trigger defense in plants (Zipfel, 2014). The remaining isolates induced less color change in cells and, correspondingly, lower levels of induced phytoalexins were detected in the extracts.

In contrast to rice cells, metabolites were not significantly elevated by simple rubbing of bacterial suspensions in 15% sucrose (OD_600_=0.2) on the leaf surface (Supplementary Fig. S5). Similarly, microbes applied on the intact leaf surface did not induce significant changes in phytohormone levels, determined by LC-MS/MS (Supplementary Fig. S6), although feeding of BPHs clearly elicited all major oxylipins (OPDA, JA, and JA-Ile), and other hormones (ABA and SA) in the leaves after insect attack (Supplementary Fig. S7). Previously, we observed induction of VOCs after combined wounding and honeydew treatments (Fig. 3), suggesting that small wounds from probing and sucking, as well as environmental damage, might be essential for microbes in honeydew to enter plant cells. We then selected the isolate 2-08, as one with low elicitor activity, and the isolate 4-24, as a strong phytoalexin inducer, to conduct the following set of combination treatments.

**Fig. 6.**
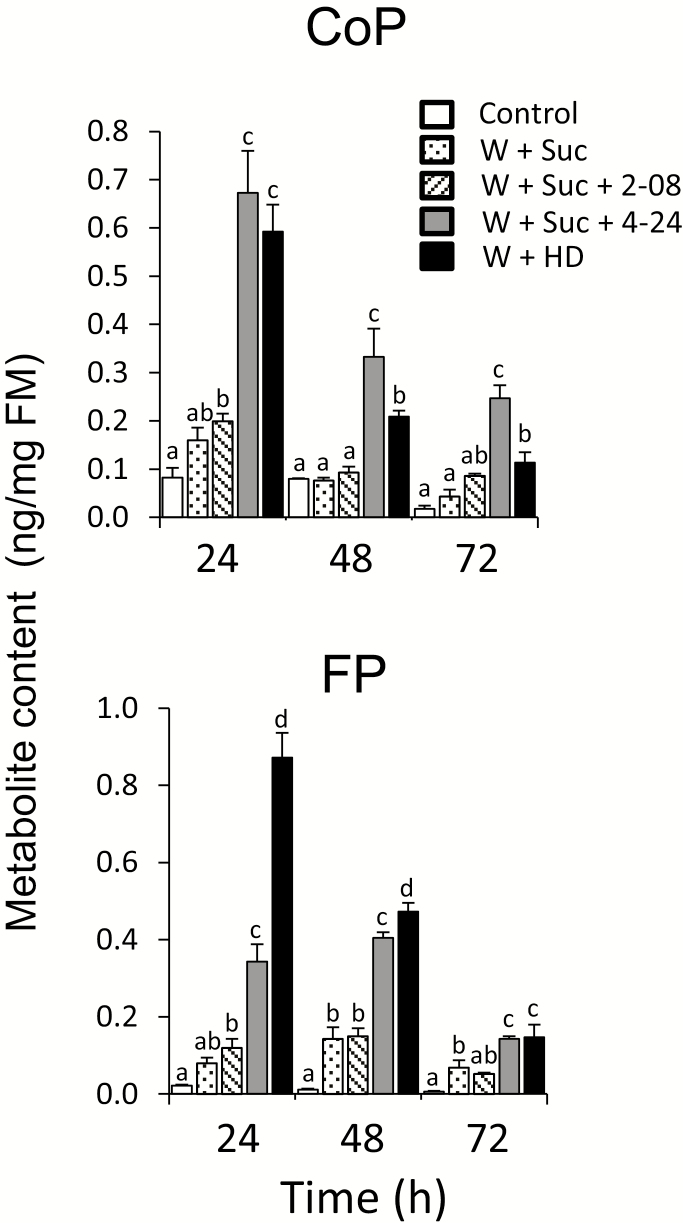
Accumulation of phytoalexins in rice leaves treated with microbial isolates from BPH honeydew. Phenolamide contents were determined in leaves after wounding with a serrated pattern wheel and applying microbial isolates suspended in 15% (w/v) sucrose adjusted to OD_600_=0.2. Sucrose and honeydew were used as negative and positive controls, respectively. Different letters show statistically significant differences between treatments by ANOVA (*P*<0.05; Tukey HSD test). *n*=4; error bars=SE; W+Suc, wounding with 15% sucrose; W+Suc+2-08, wounding with isolate 2-08 suspended in 15% sucrose; W+Suc+4-24, wounding with isolate 4-24 suspended in 15% sucrose; W+HD, wounding with raw BPH honeydew; W, wounding; Suc, sucrose; HD, honeydew; FM, fresh mass.

Bacterial isolates were suspended in 15% sucrose and, immediately after wounding with a serrated pattern wheel to mimic BPH piercing, bacterial suspensions (2 µl) were applied to the wounds. A time course of phytoalexin accumulation showed that, apart from wound-induced increases, further elevation of CoP and FP levels occurred in leaves treated with isolate 4-24. This was similar to crude honeydew application on wounded leaves (Fig. 6). At phytohormone levels, JA and JA-Ile were enhanced by crude honeydew, as well as by isolate 4-24, especially at 24–48 h post-treatment compared with wounded leaves treated with 15% sucrose as control (Supplementary Figs S8, S9). Application of isolate 2-08 showed much less effect on phenolamides and phytohormones, with levels remaining close to those of wounded leaves treated with 15% sucrose.

### Reduced phenolamide accumulation in microbe-suppressed BPHs

It is well known that microbial symbionts provide insects with essential functions, and their loss is thus detrimental to the host. This naturally obstructs unbiased tests of honeydew microbiota in the elicitation of plant defense. Nevertheless, we still attempted to obtain BPHs with suppressed microbial levels to support our findings.

**Fig. 7.**
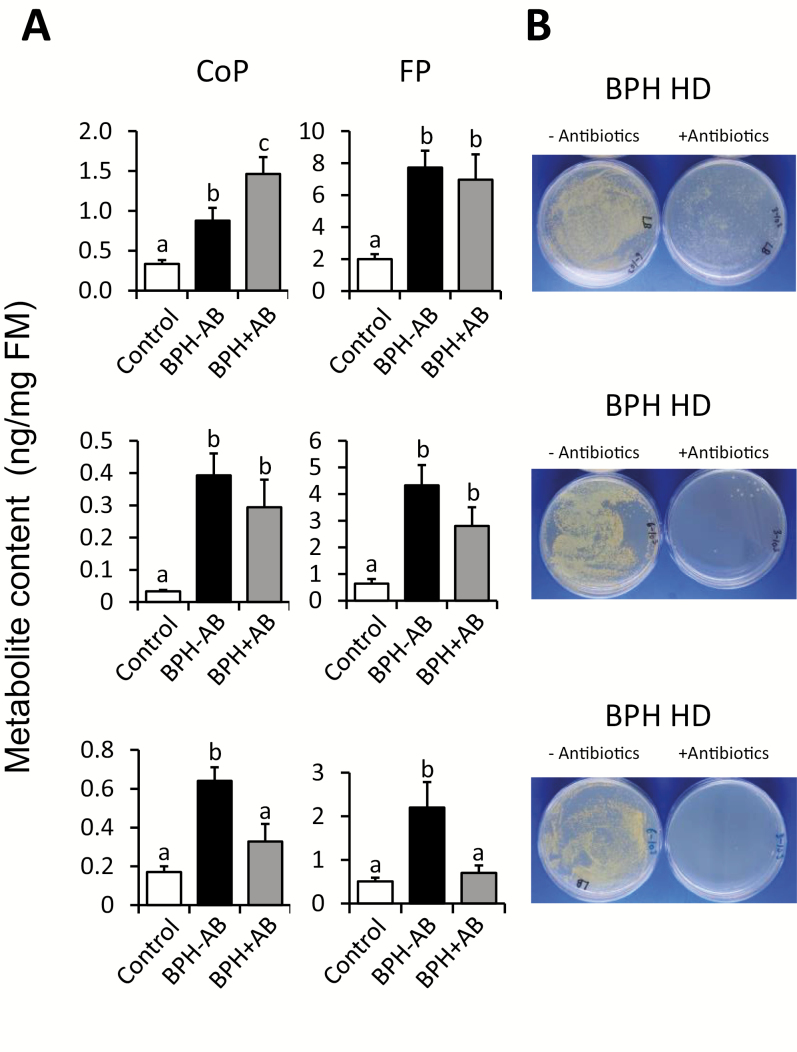
Effect of honeydew microbiota on induction of rice defense. (A) Accumulation of phytoalexins was determined in three independent experiments in rice leaves infested with BPHs pre-fed for 6 d on antibiotic- or pure water-augmented rice seedlings. Phenolamides were determined in the BPH feeding area after 4 d. Control, rice plant without BPH; BPH-AB, rice plant treated with BPHs that fed on rice seedlings without antibiotics; BPH+AB, rice plant treated with BPHs that fed on rice seedlings supplied with mixed antibiotic solution to reduce microbial flora. Different letters show statistically significant differences between treatments by ANOVA (*P*<0.05; Fisher’s HSD test). *n*=4–9; error bars=SE. (B) Honeydew released from BPHs on days 5 and 6 of the pre-treatment was collected, 1000-fold diluted in sterile water, and 50 µl was spread on LB plates. Plates are shown after 24 h incubation at 28 °C. FM, fresh mass; HD, honeydew.

Detailed information from antibiotic screening was used to design an antibiotic cocktail effective against all types of honeydew isolates, and potentially other undetermined microbes. In order to avoid further disturbance of BPHs by feeding on artificial diet, antibiotics were applied to stem cuttings prepared from young rice seedlings, and BPHs were allowed to feed on them (see Supplemental Fig. S10 for details). Method development and evaluation were carried out based on BPH mortality and residual microbe levels in the collected honeydew. For this purpose, a layer of clean Parafilm was placed under the leaves to collect honeydew from BPHs (Supplementary Fig. S10). After multiple trials, BPHs were plant-fed during an optimized 6 d cycle on antibiotic cocktails containing tetracycline (250 µg ml^–1^), rifampicin (100 µg ml^–1^), and spectinomycin (250 µg ml^–1^ (see the Materials and methods and Supplementary Fig. S10 for details). After completion of each antibiotic treatment cycle, BPHs were transferred to clip cages (4–9 biological replicates) and attached to young rice leaves for 4 d to elicit plant defense responses. Each time, a group of identically treated BPHs, but kept on seedlings without antibiotics, was used for comparison. Only leaf parts directly exposed to BPHs were collected and examined for phytoalexin levels (Fig. 7A).

In repeated trials, variable efficiency of microbe elimination in BPHs was observed (Fig. 7B). However, only when microbes were completely suppressed (bottom panel of Fig. 7B) did plants accumulate significantly less direct defense phytoalexins compared with microbe-containing BPHs (top panel in Fig. 7B), or insects that still retained the isolate 4-24 (large colonies on a plate in the central panel of Fig. 7B). Notably, isolate 4-24 showed multiple antibiotic resistances (Fig. 4B), and it was difficult to eliminate completely from BPHs. Taken together, initially observed elicitor activity of honeydew in rice, microbe effects on rice cells and plants, and antibiotic treatment results suggest that rice plants may use certain microbes from honeydew as important signals for defense. In support of this, BPH nymphs showed higher mortality between 2 d and 4 d of feeding exposure to crude honeydew-treated leaves compared with filtered honeydew application (Supplementary Fig. S11).

## Discussion

Plants perceive various signals from the environment, which inform them about changes, and potential danger, such as the presence of pathogens and herbivores (Gust *et al.*, 2017). Accordingly, we show that microbes from honeydew of BPHs can effectively elicit rice defenses. The antibiotics fed to BPHs suppressed cultivable microbes in the honeydew, which in turn attenuated the inducible defenses of rice against BPHs. We propose a model in which microbes in honeydew are perceived by rice to amplify its defense, which acts in addition to elicitors found in the BPH salivary secretions, and mechanical damage of various sources.

### Quest for sucking/piercing arthropod elicitors

In contrast to chewing herbivores, piercing/sucking arthropods, such as BPHs used in this study, are associated with a minimal level of mechanical injury (Walling, 2008), which can be further masked by effectors released from the plant intruders (Bos *et al.*, 2010; Atamian *et al.*, 2013; Rodriguez *et al.*, 2014; Naessens *et al.*, 2015; Villarroel *et al.*, 2016). For example, salivary EF-hand calcium-binding protein NlSEF suppresses defense responses in rice against BPHs (Ye *et al.*, 2017). Another secreted salivary effector, endo-β-1,4-glucanase NlEG1, enables the stylet of BPHs to reach phloem cells by degrading cellulose in the protective plant cell walls (Ji *et al.*, 2017). Reports by Ye *et al.* (2017) and Ji *et al.* (2017), and findings of Petrova and Smith (2015), suggest that BPHs are involved in reprograming of the rice transcriptome, leading to nutritional enhancements that benefit them. However, in spite of the effectors, rice still vigorously responds to feeding of BPHs by accumulation of phytoalexins (Alamgir *et al.*, 2016; insets in Fig. 2), and activates its hormonal signaling (Supplementary Fig. S7). Therefore, one or more dominant elicitors from BPHs must be perceived by rice.

Recently, secreted mucin-like protein from the salivary sheets of BPHs was shown to trigger defense responses in rice, including cell death, expression of defense-related genes, and callose deposition (Shangguan *et al.*, 2018). In our report, honeydew, and specifically honeydew-associated microbes, elicited chemical defenses, such as phytoalexins, and release of VOCs from the rice plants. While honeydew is rich in nutrients, and thus widely open to contaminations, the microbes can originate from various sources, including plant interiors (e.g. phloem-restricted bacteria), the BPH digestive tract (e.g. insect gut symbionts), or the environment. From the functional perspective, insect-associated symbionts appear to be particularly good targets for evolution of novel signals of defense in plants (Schausberger, 2018). These can be delivered to plants via honeydew as shown here, or via saliva secreted to phloem during BPH feeding.

### Insect-associated microorganisms modulate plant defense

In this study, BPH honeydew contained seven strains of culturable bacteria (Table 1; Supplementary Table S1). From the BPH genome and its analysis, yeast-like symbionts (YLSs) also occur in BPHs which complements essential nutritional pathways in the planthopper (Chen, 1981; Tang *et al.*, 2010; Xue *et al.*, 2014). However, YLSs have not been found on LB and GAM media, possibly due to their non-culturable character, or lack of their secretion into honeydew, and their role in plant defense needs to be examined separately.

Addressing the commonality of microbes we isolated from honeydew, six of them were previously reported as gut microbial symbionts of BPH (*Acinetobacter* in Tang *et al.*, 2010; *Acinetobacter*, *Staphylococcus*, and *Serratia* in Wang *et al.*, 2015; and *Acinetobacter*, *Serratia*, *Microbacterium*, and *Corynebacterium* in Malathi *et al.*, 2018). In addition, endosymbionts determined by 16S rRNA gene amplicon sequencing in the small brown planthopper (*Laodelphax striatellus* Fallén) included bacterial genera *Staphylococcus*, *Acinetobacter*, *Microbacterium*, and *Corynebacterium* (Li *et al.*, 2017) also found in our study. In particular, *Serratia* and *Acinetobacter* seem to be associated with various insects: *Serratia* was found in locusts (Dillon *et al.*, 2002), squash-bugs (Bruton *et al.*, 2003; Wayadande *et al.*, 2005), houseflies (Cooke *et al.*, 2003), crickets (Adamo, 2004), Formosan termites (Adams and Boopathy, 2005), peach potato aphids (Saguez et al., 2005), diamondback moth (Indiragandhi *et al.*, 2007), ground beetle (Lundgren *et al.*, 2007), pecan phylloxera (Medina *et al.*, 2011), and western tarnished plant bug (Cooper *et al.*, 2014). *Acinetobacter* was found in diamondback moth (Indiragandhi *et al.*, 2007), *Dactylopius* spp. (Ramirez-Puebla et al., 2010), and mosquito vector (Zouache, 2009). While the role of gut symbionts may vary in each insect, they are generally required for maintaining a suitable gut environment (Adams and Boopathy, 2005; Medina *et al.,* 2011) and production of enzymes such as chitinases by *Serratia* and *Acinetobacter*, and they contribute to host nutrition (Whitaker *et al.,* 2004; Ruiz-Sanchez *et al., *2005; Indiragandhi *et al., * 2007), for example by supplementing essential amino acids that are lacking in plant sap (Lamelas *et al.,* 2008).

Numerous benefits for herbivores from the presence of gut bacteria have possibly counterbalanced the negative selection pressure implied from their function as alarm signals against their herbivore host in plants, when living bacteria or their residues are involuntarily deposited on plants as part of the insect secretions, honeydew and/or saliva. In BPHs, both honeydew and honeydew-isolated microbes, including gut symbionts *Acinetobacter soli* (2-19) and *Serratia marcescens* subsp. *marcescens* (4-24) strongly elicited phytoalexin levels in rice (Figs 2, 6). Interestingly, not all microbes from honeydew were able to amplify herbivory and/or wound-induced defense in rice, like another gut bacteria previously found in BPH, *Staphylococcus sciuri* (2-06). Regarding the observed selectivity, we still need to determine how these microbes are discriminated by rice.

### Microbial effects on direct and putative indirect defenses

We show that both honeydew and microbes induced phenolamide phytoalexins that serve as direct defense against BPHs in rice (Alamgir *et al.*, 2016). VOCs are also rapidly elicited during herbivore attack to recruit natural enemies of herbivores (Aljbory and Chen, 2018), which constitutes a sophisticated system of indirect plant defense. Although various microbe-derived VOCs affect plant growth and defense (Bitas *et al.*, 2013; Junker and Tholl, 2013; Liu and Zhang, 2015), much less is known about the microbe-induced production of volatiles in plants. Here, we found that VOCs from rice can be positively modulated by microbe-containing BPH honeydew application. As direct and indirect defense responses are well known to suppress insect performance ((Mithöfer and Boland, 2012), phenolamides and VOCs elicited by honeydew (and its microbiota) in this study are expected to reduce the performance of BPHs adequately under natural conditions.

Induction of two pathways, direct (phenolamides) and indirect (VOCs) defense, suggests that honeydew affects some early steps in the rice defense cascade, such as phytohormone levels, and/or downstream signal transduction mechanisms (Wasternack and Song, 2017). While we have already examined phytohormones, it is still necessary to investigate other components of plant defense signaling against herbivores, such as Ca^2+^-mediated responses (Arimura and Maffei, 2010), levels of reactive oxygen species (ROS) (Zebelo and Maffei, 2015; Shinya et al., 2016), or activity of mitogen-activated protein (MAP) kinases (Hettenhausen *et al.*, 2015). Finally, transcription factors induced by honeydew, and its components, that directly link signaling to defense genes, should be taken into consideration (Woldemariam *et al.*, 2011).

### Hormonal pathways under attack

Defense responses against sucking insects, including BPHs, resemble pathogen-induced alterations (Walling, 2000; Zhou *et al.*, 2009; Duan *et al.*, 2014; Villarroel *et al.*, 2016). Here, complexity arises from the antagonism in plant defense signaling against insects and pathogens, which is mediated by JA and SA, respectively (Glazebrook, 2005). For example, feeding of whiteflies induced SA, which in turn suppressed jasmonate-mediated responses in Arabidopsis (Zhang *et al.*, 2013). Some chewing insects use microbes and SA to subdue wound- and HAMP-elicited jasmonate signaling (Chung *et al.*, 2013*b*). Similarly, honeydew from aphids suppressed JA signaling via the salicylate pathway in broad bean plants (*Vicia faba*), partly via SA contained in honeydew (Schwartzberg and Tumlinson, 2014). Whiteflies even glycosylate SA, and deploy this conjugate to counteract plant defense (VanDoorn *et al.*, 2015). A whitefly-associated facultative symbiont *Hamiltonella defense* that occurs in salivary secretions also suppressed induced defense responses in tomato (Su *et al.*, 2015).

Although microbes and SA are helping invaders to subdue plant defenses against herbivores, BPH honeydew and its microbes clearly worked as amplifiers of wound-induced rice defense. We also report that feeding of BPHs elicits high levels of JA, and its active form JA-Ile, after 1 d and 3 d, respectively, while SA levels only increased significantly after 4 d of BPH feeding. It can be assumed that rice is using more instant information from the honeydew to elicit JA signaling before substantial changes in SA levels can take place (Supplementary Fig. S7). However, caution is needed as rice contains extraordinary amounts of constitutive SA (Supplemental Fig. S7), and it is not clear which fraction of total extracted SA is active in cell defense and metabolism.

Similar to our study, elimination of bacterial symbionts from herbivores to study hormonal responses was attempted before. Reduced microbial levels in herbivorous spider mites *Tetranychus urticae* affected pest performance but this was not linked to significant changes in JA-Ile levels in the host plants (Staudacher *et al.*, 2017). Interestingly, OPDA precursors of JA were enhanced and suppressed in the presence of two microbes, *Wolbachia* and *Spiroplasma*, in the spider mites, respectively (Staudacher *et al.*, 2017). In contrast, OPDA levels correlated with the increases of JA and JA-Ile in rice leaves infested with BPHs in our study. Not only BPHs but also honeydew and bacterial isolate 4-24 promoted JA and JA-Ile levels in the wounded leaves of rice (Supplementary Figs S8, S9). Somewhat in contradiction to our results, silencing of Os*HI-LOX* (lipoxygenase), a gene involved in JA biosynthesis in rice, suppressed resistance to chewing insects but induced tolerance responses to BPHs under laboratory/greenhouse conditions (Zhou *et al.*, 2009). Perhaps future experiments under natural conditions, including access to tritrophic interactions, will support the positive role of JA in defense signaling against BPHs, and highlight the importance of microbe- and honeydew-promoted direct and indirect defense responses found in this study.

## Supplementary data

Supplementary data are available at *JXB* online.

Fig. S1. BPH-infested plants in the field and honeydew-induced secondary metabolite contents in rice cells.

Fig. S2. Phenolamide contents in rice seedlings treated with BPH honeydew.

Fig. S3. Induction of phytoalexins in rice cells treated with honeydew fractions.

Fig. S4. Induction of phytoalexins in rice cells treated with filtered and/or heated honeydew.

Fig. S5. Accumulation of phytoalexins in intact rice plants treated with microbial isolates from BPH honeydew.

Fig. S6. Accumulation of phytohormones in intact rice leaves treated with microbial isolates suspended in 15% (w/v) sucrose.

Fig. S7. Phytohormone accumulation in rice leaves infested with BPH.

Fig. S8. Accumulation of phytohormones in wounded rice leaves treated with BPH honeydew.

Fig. S9. Accumulation of phytohormones in wounded rice leaves treated with microbial isolates suspended in sucrose.

Fig. S10. Custom-design system for treatment of BPH adults with antibiotics.

Fig. S11. Performance of BPH on crude and filtered honeydew-treated leaves.

Table S1. Identification of microbes isolated from BPH honeydew in DDBJ/EMBL.

Supplementary Table S1 and Figures S1-S11

## Abbreviations:

ABA: abscisic acid
BPH: brown planthopper
CoP
FA: , *N*-acetylchitooctaose
FP: feruloylputrescine
HAMP: herbivore-associated molecular pattern
JA: jasmonic acid
JA-Ile: jasmonoyl-l-isoleucine
OPDA: 12-oxo-phytodienoic acid
PAMP: pathogen-associated molecular pattern
SA: salicylic acid
VOC: volatile organic compound.
