# Supplementary material for: Honeydew-associated microbes elicit defense responses against brown planthopper in rice
Source: J Exp Bot. 2019 Mar 2;70(5):1683–96. doi: 10.1093/jxb/erz041 (PMC6411376; doi:10.1093/jxb/erz041)
Supplement: Supplementary Table S1 and Figures S1-S11 [file erz041_suppl_supplementary_table-s1_and_figures_s1-s11.pdf]

Supplementary Data

Honeydew-associated microbes elicit defense responses against brown planthopper in rice

David Wari[1], Kabir Md Alamgir[1], Kadis Mujiono[1,2], Yuko Hojo[1], Tomonori Shinya[1], Akio Tani[1], Hiroko Nakatani[1], Ivan Galis[1] *

[1]Institute of Plant Science and Resources, Okayama University, Kurashiki 710-0046, Japan

[2] Faculty of Agriculture, Mulawarman University, Samarinda 75119, Indonesia

*Corresponding author: igalis@okayama-u.ac.jp

Supplementary Tables

Table S1. **Identification of microbes isolated from BPH honeydew in DDBJ/EMBL.** The isolates were further compared to DDBJ and EMBL databanks for identification of clones previously isolated from BPH. In the blast search, sequences highly similar to isolates 2-06, 2-19 and 4-24 were previously reported as endosymbionts of BPH (*Nilaparvata lugens*).

DDBJ/EMBL Blast Search				
Isolate	Blast hit	Similarity (%)	Accession number	Reference
2-06	Endosymbiont of *Nilaparvata lugens*	99	JQ975877	Wang et al. 2015
2-08	*Staphylococcus xylosus*	99	LN554884	Loux, 2014
2-19	Endosymbiont of *Nilaparvata lugens*	99	GU124492	Wang et al. 2015
3-16	*Pantoea dispersa*	99	AB907780	Tanaka et al. 2015
4-05	*Microbacteriaceae laevaniformans*	99	EU879962	Bernard and Munro, 2008
4-07	*Corynebacterium glyciniphilum*	99	CP006842	Al-Dilaimi et al. 2013
4-24	Endosymbiont of *Nilaparvata lugens*	99	GU124498	Wang et al. 2015

Cited references

Wang W, Zhu T, Lai F, Fu Q. 2015. Diversity and infection frequency of symbiotic bacteria in different populations of the rice brown planthopper in China. Journal of Entomological Science **50**, 47-66.

Loux V. 2014. https://www.ncbi.nlm.nih.gov/nuccore/LN554884

Kato Tanaka Y, Horie N, Mochida K, Yoshida Y, Okugawa E, Nanjo F. 2015. *Pantoea theicola* sp. nov., isolated from black tea. International Journal of Systematic and Evolutionary Microbiology **65**, 3313-3319.

Bernard K, Munro C. 2008. https://www.ncbi.nlm.nih.gov/nuccore/EU879962

Al-Dilaimi A, Bednarz H, Lömker A, Niehaus K, Kalinowski JR, Rückert C. 2015. Revisiting *Corynebacterium glyciniphilum* (ex Kubota et al., 1972) sp. nov., nom. rev., isolated from putrefied banana. International Journal of Systematic and Evolutionary Microbiology **65**, 177-182.

Supplementary Figures

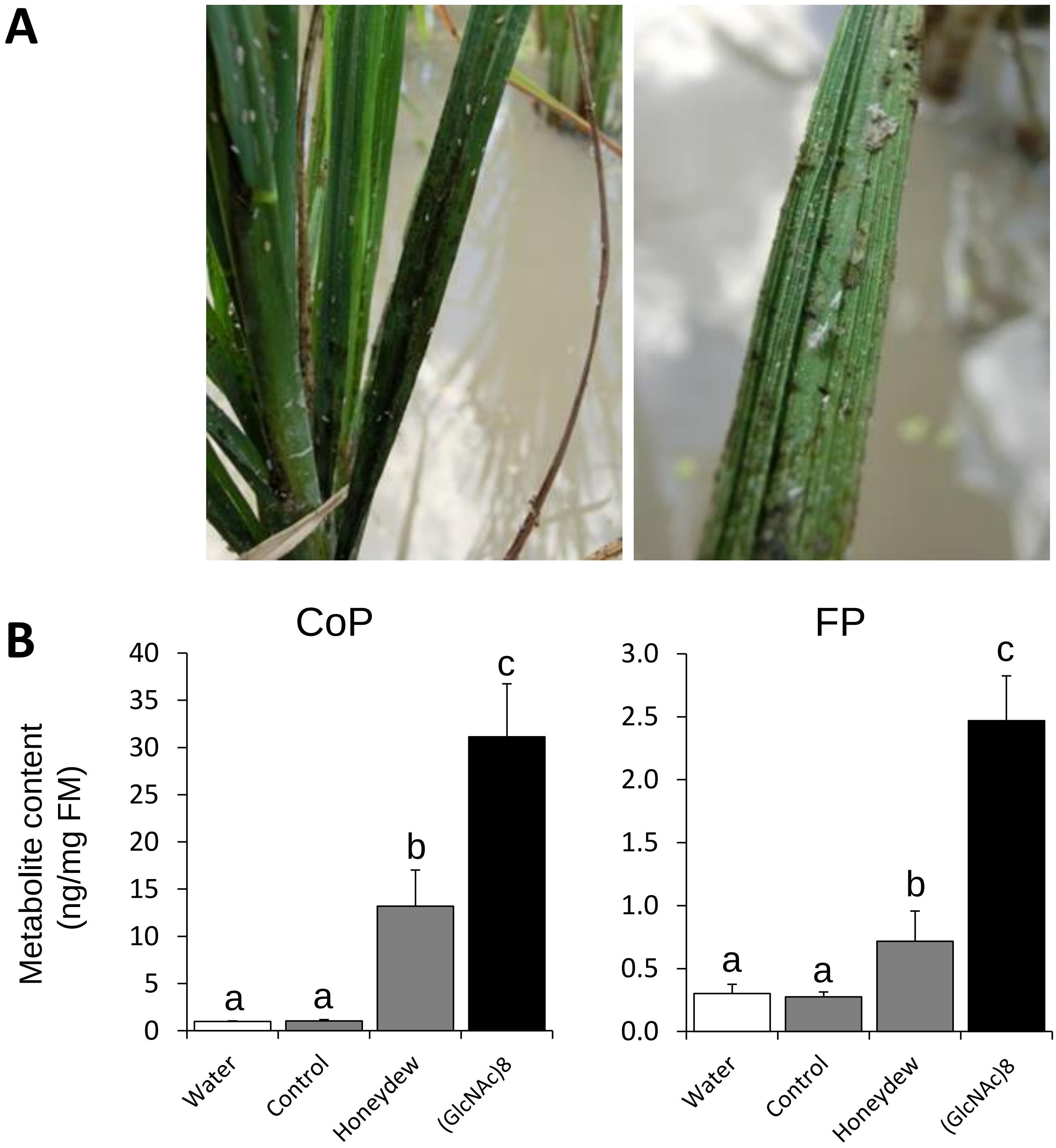

Figure S1. **BPH infested plants in the field and honeydew induced secondary metabolite contents in the rice cells.** (A) Field-grown rice plants infested with planthoppers show sooty phenotype caused by honeydew deposition, fungal growth, and other microbes. (B) Cultured rice cells were treated with honeydew and incubated for 24 h before extraction and determination of phenolamide contents by LC-MS/MS. Water and chitin oligomer $(GlcNAc)_8$ were used as negative and positive control treatments, respectively. Letters indicate statistically significant differences between treatments determined by ANOVA followed by Tukey HSD test ($P<0.05$). n=4, water and $(GlcNAc)_8$; n=8, control (no-BPH clip cage wash) and honeydew; error bars = SEM; FM, fresh mass.

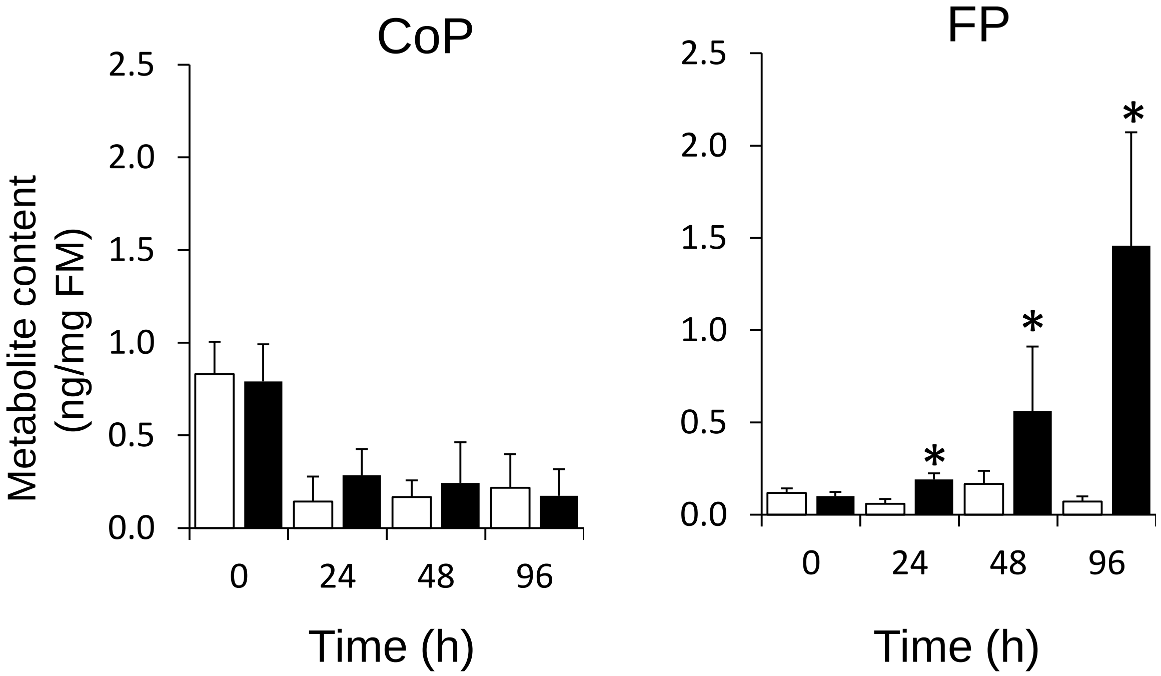

Figure S2. **Phenolamide contents in rice seedlings treated with BPH honeydew.** Young rice seedlings of approximately 4 weeks age were treated with honeydew on the leaves and sampled every 24 h for four days. Leaves were extracted and phenolamide levels were determined by LC-MS/MS. Asterisks indicate statistically significant differences between treatment and control of the respective time point, determined by Student's t-test at $P<0.05$. n=3, error bars = SEM; FM, fresh mass.

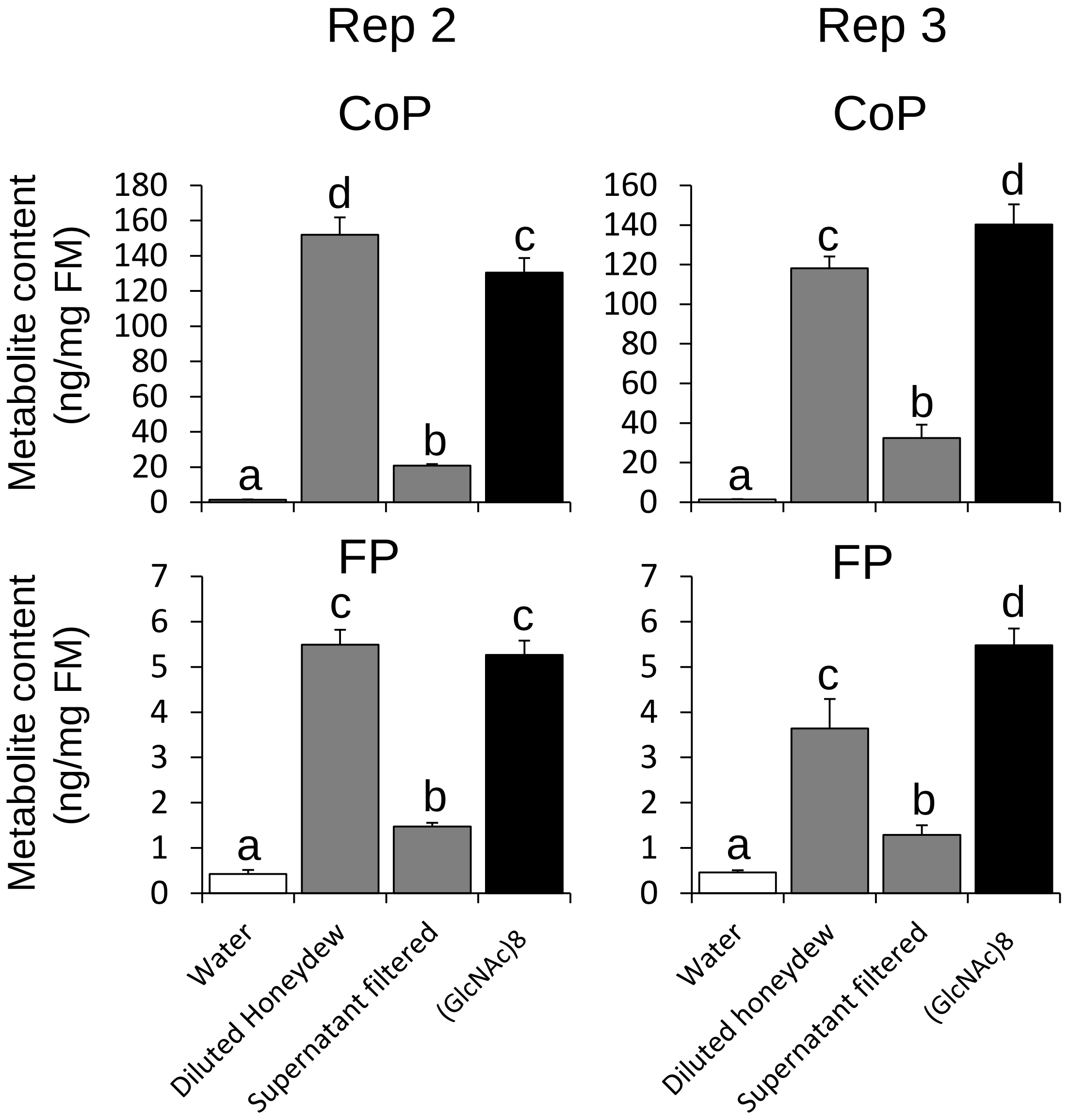

Figure S3. **Induction of phytoalexins in rice cells treated with honeydew fractions.** Rice cells were treated for 24 h with filtered BPH honeydew supernatant, diluted honeydew, and chitin oligomer $(GlcNAc)_8$ as positive control in two additional separate experiments from data shown in Fig. 4A. Different letters show statistically significant differences between treatments by ANOVA (*P*<0.05; Tukey HSD test). n=3; error bars = SEM; FM, fresh mass.

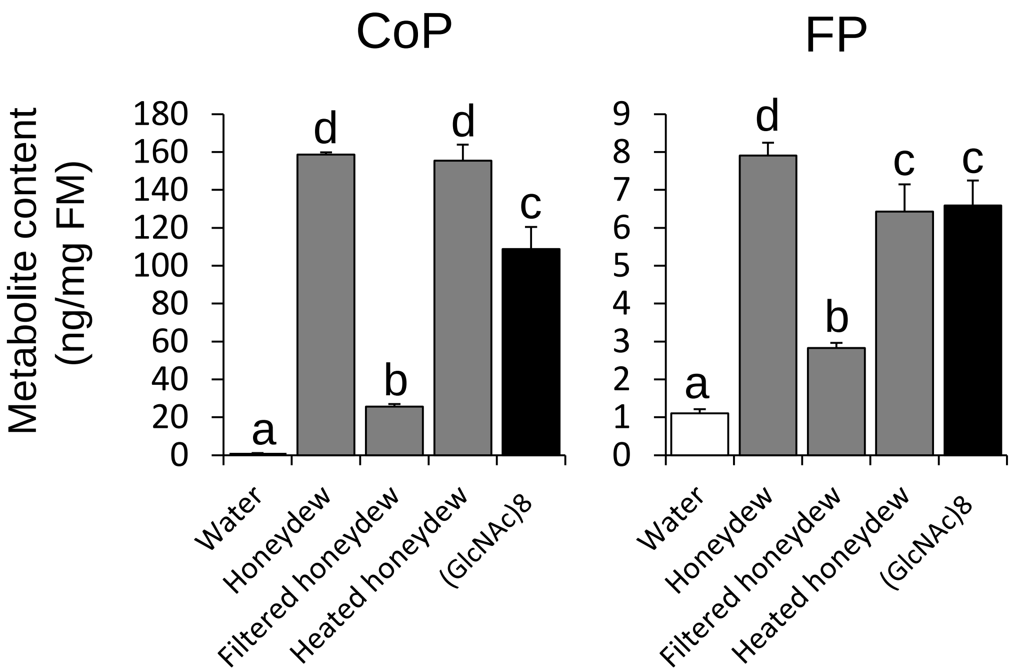

Figure S4. **Induction of phytoalexins in rice cells treated with filtered and/or heated honeydew.** Rice cells were treated with raw and filtered BPH honeydew, heat-treated honeydew, and positive control chitin oligomer $(GlcNAc)_8$ for 24 h. Notably, heat treatment did not abolish honeydew activity but filtration was highly effective. Different letters show statistically significant differences between treatments by ANOVA ($P<0.05$; Tukey HSD test); n=3; error bars = SEM; FM, fresh mass.

Fig S5

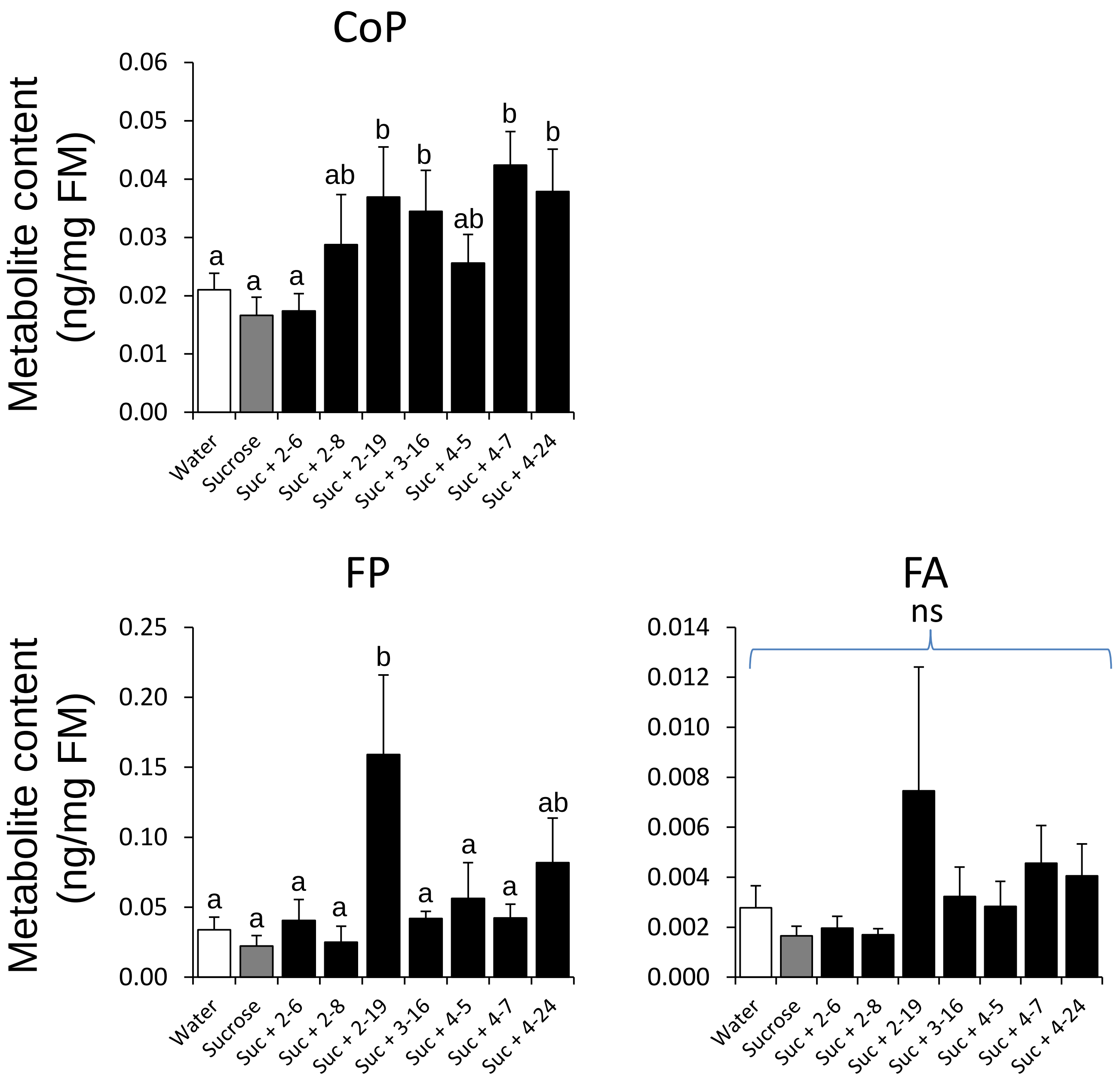

Figure S5. **Accumulation of phytoalexins in intact rice plants treated with microbial isolates from BPH honeydew.** Rice leaves after rubbing on surface the microbial isolates suspended in 15% (w/v) sucrose in sterile water were incubated for 3 d. After harvesting, leaves were extracted and metabolites were determined by LC-MS/MS. Different letters show statistically significant differences between treatments by ANOVA (*P*<0.05; Tukey HSD test); n=4; error bars = SEM; FM, fresh mass; Suc, Sucrose; ns, not significant.

Fig S6

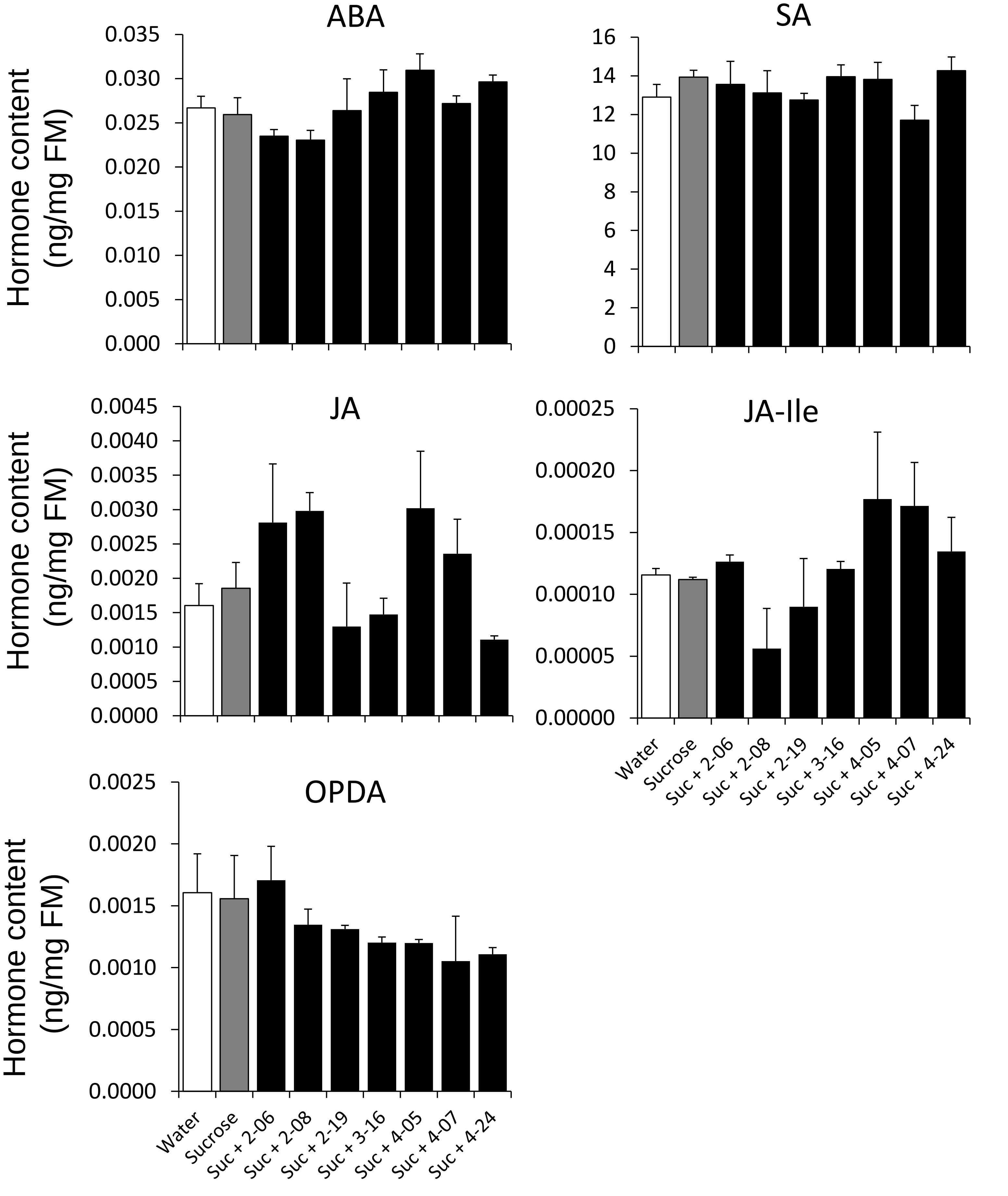

Figure S6. **Accumulation of phytohormones in intact rice leaves treated with microbial isolates suspended in 15% (w/v) sucrose.** Abscisic acid (ABA), salicylic acid (SA), jasmonic acid (JA), jasmonoyl-L-isoleucine (JA-Ile), and 12-oxo-phytodienoic acid (OPDA) levels were determined in the leaves 72 h after rubbing treatment with microbial isolates from BPH honeydew (OD_{600} = 0.2). No statistically significant differences were observed between treatments by ANOVA (*P*<0.05; Tukey HSD test); n=4; error bars = SEM; FM, fresh mass. OPDA is expressed as equivalent of d3-JA.

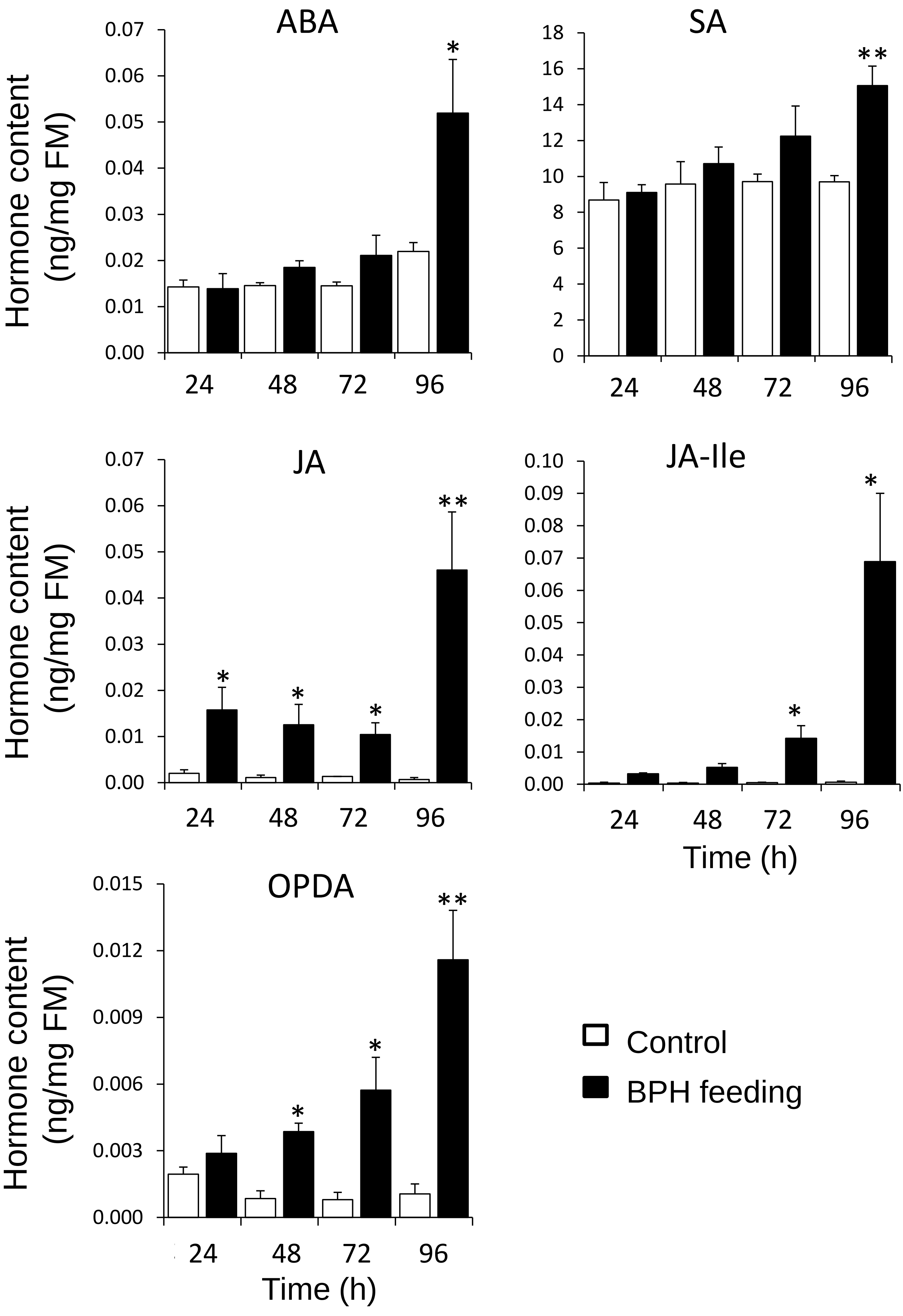

Figure S7. **Phytohormone accumulation in rice leaves infested with BPH.** Abscisic acid (ABA), salicylic acid (SA), jasmonic acid (JA), jasmonoyl-L-isoleucine (JA-Ile), and 12-oxo-phytodienoic acid (OPDA) levels were determined in the leaves after attaching 10 BPH adults in clip cage to the youngest fully developed leaf on the rice plant. Asterisks indicate statistically significant differences between treatments determined at each time point by Student's t-test (**P*<0.05; ** *P*<0.01); n=5; error bars = SEM; FM, fresh mass. OPDA is expressed as equivalent of d3-JA.

Fig S8

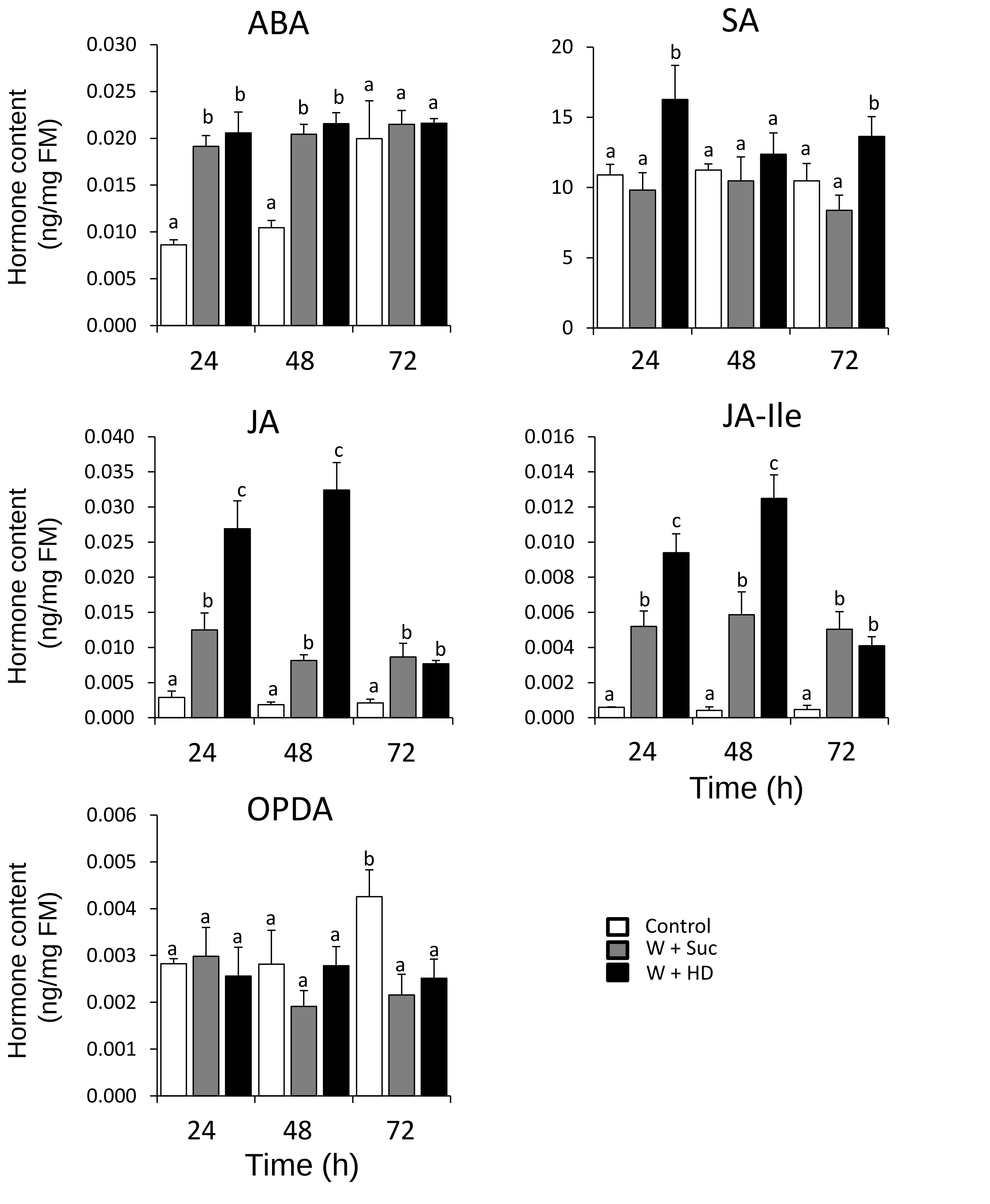

Figure S8. **Accumulation of phytohormones in wounded rice leaves treated with BPH honeydew.** Rice leaves were wounded with a pattern wheel on both sides of the lamina and honeydew was rubbed on the leaves. Samples were collect every 24 h up to 4 d and used for extraction and determination of abscisic acid (ABA), salicylic acid (SA), jasmonic acid (JA), jasmonoyl-L-isoleucine (JA-Ile), and 12-oxo-phytodienoic acid (OPDA) levels by LC-MS/MS. Different letters show statistically significant differences between treatments by ANOVA (P<0.05; Tukey HSD test); n=4; error bars = SEM; FM, fresh mass; W+Suc, wounding with 15% sucrose; W+HD; wounding with BPH honeydew. OPDA is expressed as equivalent of d3-JA.

Fig S9

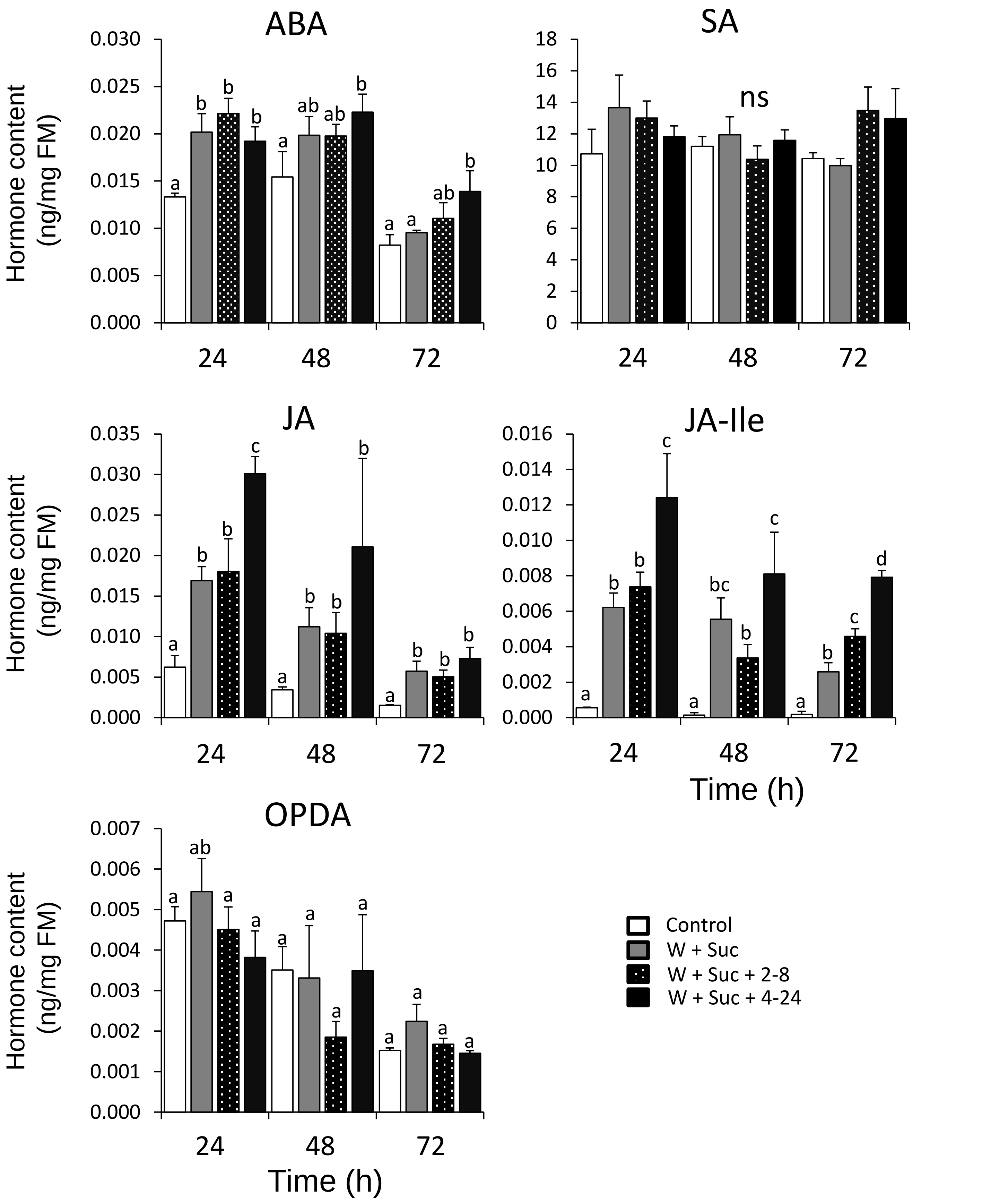

Figure S9. **Accumulation of phytohormones in wounded rice leaves treated with microbial isolates suspended in sucrose.** Rice leaves were wounded with a pattern wheel on both sides of the lamina and microbial isolates (OD_{600} = 0.2) suspended in 15% sucrose solution were rubbed on the leaves. Samples were collect after 24 h and used for extraction and determination of abscisic acid (ABA), salicylic acid (SA), jasmonic acid (JA), jasmonoyl-L-isoleucine (JA-Ile), and 12-oxo-phytodienoic acid (OPDA) levels by LC-MS/MS. Different letters show statistically significant differences between treatments by ANOVA (P<0.05; Tukey HSD test); n=4; error bars = SEM; FM, fresh mass. W+Suc, wounding with 15% sucrose; W+Suc+2-08, wounding with isolate 2-08 suspended in 15% sucrose; W+Suc+4-24, wounding with isolate 4-24 suspended in 15% sucrose. OPDA is expressed as equivalent of d3-JA.

Fig S10

A

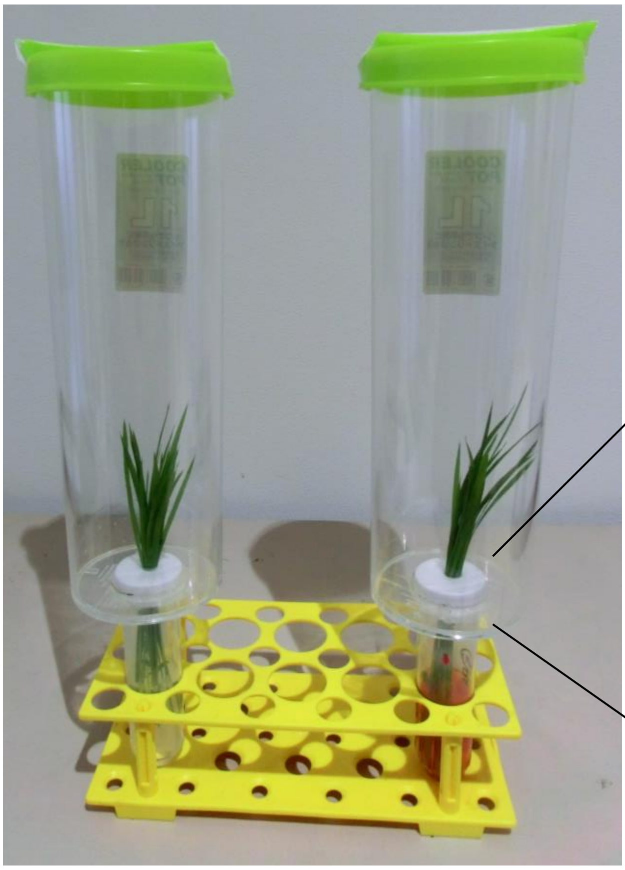

Antibiotic	Concentration
Tetracycline	250 µg/mL
Rifampicin	100 µg/mL
Spectinomycin	250 µg/mL

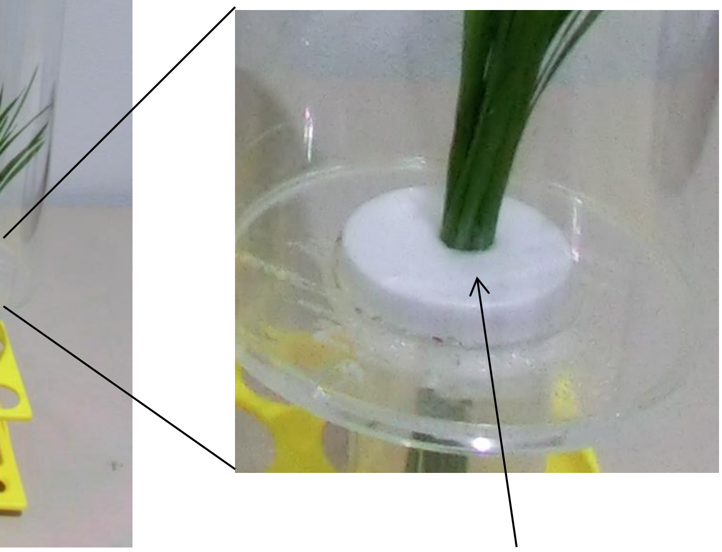

B

	Cut leaves with AB	Transfer BPH	
Day -1	PM (o/n)		
Day 0	PM (o/n)	AM	
Day +1	PM (o/n)	AM	
Day +2	PM (o/n)	AM	
Day +3	PM (o/n)*	AM	
Day +4		AM	
Day +5			
Day +6	Set BPH on plants; collect HD from Parafilm		→ Bacteria elimination test on LB plate
Day +7			
Day +8			
Day +9			
Day +10	Collect leaves for analysis		

* AB concentration reduced to 1/5

Figure S10. **Custom-design system for treatment of BPH adults with antibiotics.** (A) Young rice seedling were cut and placed in sterile water with antibiotics or water (control). (B) Schedule used for preparation of antibiotic containing seedlings and transfer schedule of BPHs to freshly prepared containers with plants. PM, leaves were cut and inserted in antibiotic solution in the afternoon; AM, BPH were transferred to pre-incubated leaves; o/n, incubation over night; AB, antibiotic mix; HD, honeydew.

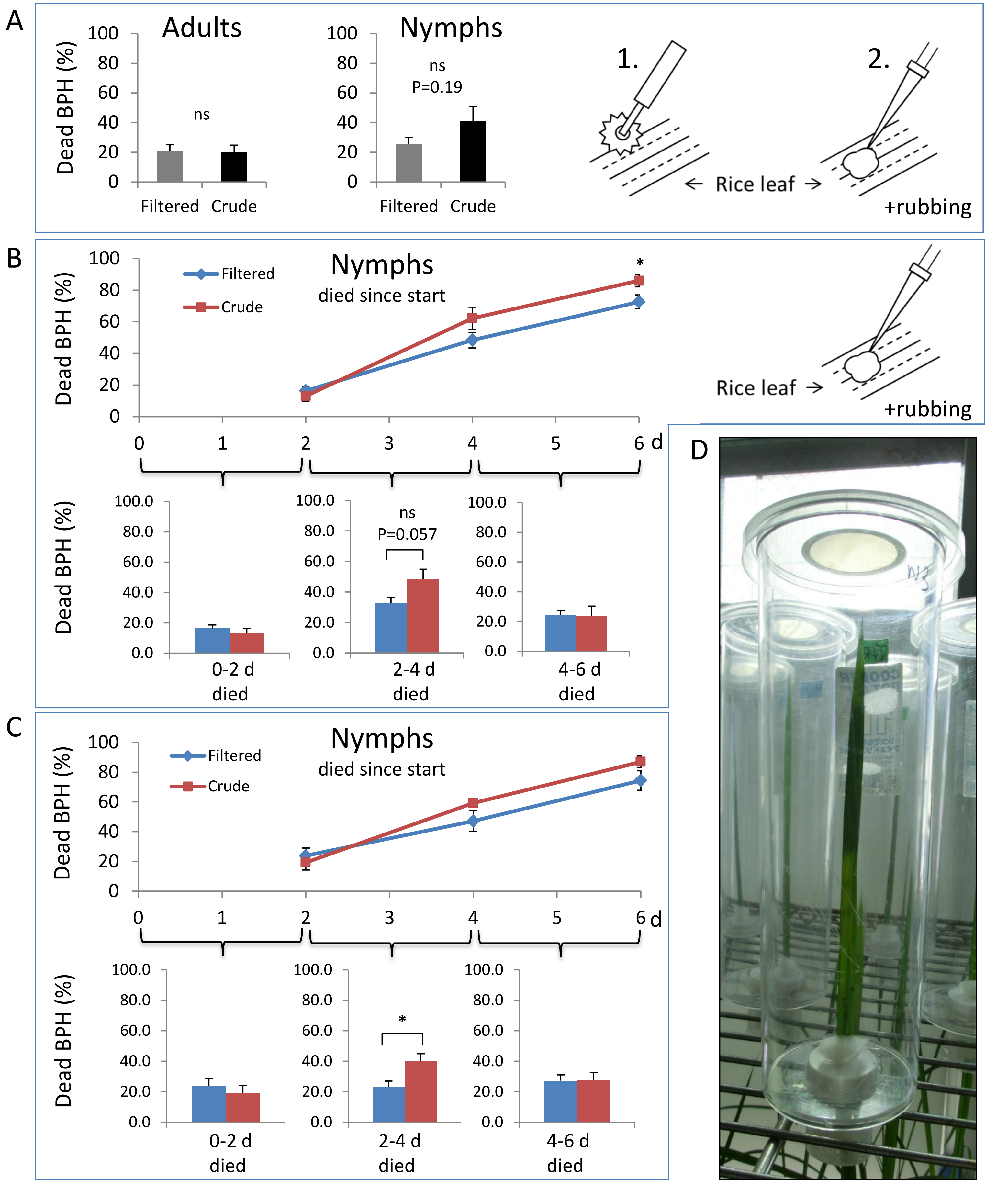

Figure S11. **Performance of BPH on crude and filtered honeydew-treated leaves.** (A) Youngest leaf of rice seedlings was wounded with a pattern wheel and 5 µL of crude or filtered honeydew was gently rubbed on leaf surface. 10 adults BPH (n=5) or 15 nymphs (n=8) were set on each leaf contained in cylinder after 1 d, and survival rate was observed after 7 d. (B) Unwounded plants were gently rubbed to apply 10 µL of crude or filtered honeydew. 15 BPH 3-4 instar nymphs were set on each leaf contained in cylinder after 3 d (n=8). Dead BPHs were counted and removed every 2 days. Breakdown of dead BPH nymphs in each 2-day-interval are shown in bar graphs. (C) Repeat of experiment shown in B. (D) Experimental setup. Consistently, more dead BPH nymphs tend to be recovered between 2-4 d in plants treated with crude compared to filtered honeydew. Asterisks show significant difference between treatments determined by Student t-test (*P<0.05). ns, not significant.
